# Endolysosomal TRPML1 channel regulates cancer cell migration by altering intracellular trafficking of E-cadherin and β_1_-integrin

**DOI:** 10.1016/j.jbc.2023.105581

**Published:** 2023-12-21

**Authors:** Nadine Frey, Lina Ouologuem, Julia Blenninger, Wei-Xiong Siow, Julia Thorn-Seshold, Jan Stöckl, Carla Abrahamian, Thomas Fröhlich, Angelika M. Vollmar, Christian Grimm, Karin Bartel

**Affiliations:** 1 Department of Pharmacy, Pharmaceutical Biology, Ludwig-Maximilians-University Munich, Munich, Germany; 2 Department of Pharmacy, Ludwig-Maximilians-University Munich, Munich, Germany; 3 Gene Center, Laboratory for Functional Genome Analysis, Ludwig Maximilians-University Munich, Munich, Germany; 4 Walther-Straub-Institute of Pharmacology and Toxicology, Ludwig-Maximilians-University Munich, Munich, Germany

**Keywords:** cancer biology, migration, adhesion, lysosome, ion channel

## Abstract

Metastasis still accounts for 90% of all cancer-related death cases. An increase of cellular mobility and invasive traits of cancer cells mark two crucial prerequisites of metastasis. Recent studies highlight the involvement of the endolysosomal cation channel TRPML1 in cell migration. Our results identified a widely antimigratory effect upon loss of TRPML1 function in a panel of cell lines *in vitro* and reduced dissemination *in vivo*. As mode-of-action, we established TRPML1 as a crucial regulator of cytosolic calcium levels, actin polymerization, and intracellular trafficking of two promigratory proteins: E-cadherin and β_1_-integrin. Interestingly, KO of TRPML1 differentially interferes with the recycling process of E-cadherin and β_1_-integrin in a cell line–dependant manner, while resulting in the same phenotype of decreased migratory and adhesive capacities *in vitro*. Additionally, we observed a coherence between reduction of E-cadherin levels at membrane site and phosphorylation of NF-κB in a β-catenin/p38-mediated manner. As a result, an E-cadherin/NF-κB feedback loop is generated, regulating E-cadherin expression on a transcriptional level. Consequently, our findings highlight the role of TRPML1 as a regulator in migratory processes and suggest the ion channel as a suitable target for the inhibition of migration and invasion.

Despite significant advances in cancer therapy (1, 2), cancer remains one of the main causes of death worldwide (3). Owing to still limited therapy options, 90% of the cancer-related death cases are correlated to metastasizing tumorigenic cells. Tumor metastasis is initiated by increasingly invasive primary tumor cells migrating into the surrounding tissue and subsequently penetrating blood or lymphatic vessels. Upon reaching the secondary tumor site, cells extravagate and proliferate within the organ leading to the growth of the metastatic tumor (4, 5).

A prerequisite for metastasis is the increase in cellular mobility (6). Cells migrate either individually (amoeboid and mesenchymal migration) or collectively as a group of cells (7). This movement is predominantly regulated by two classes of adhesion proteins—cadherins facilitating cell–cell adhesion and integrins mediating cell-matrix adhesion (8, 9, 10, 11). In this context, especially epithelial-cadherin (E-cadherin) has become the scope of intense research. E-cadherin is a calcium-dependent single-pass transmembrane glycoprotein that mediates cell–cell adhesion through homotypic binding with neighboring cells (12, 13). E-cadherin is stabilized at the cellular membrane by p120-catenin and linked to the actin cytoskeleton through β- and α-catenin, allowing the regulation of actin polymerization (14). Despite its established status as tumor suppressor, the role of E-cadherin in cancer is ambivalent (14). On the one hand, loss of E-cadherin is associated with highly invasive cancers as it allows individual dissemination from the tumor promoting individual mesenchymal migration—a process summarized as the epithelial-to-mesenchymal transition (EMT) (15). On the other hand, E-cadherin can be retained in some types of cancer like colon carcinoma as a regulator of the collective cell migration. As such, it aids maintaining strong intercellular contacts, which mediate the mechanotransduction required for migration (16, 17).

The family of integrins, heterodimeric transmembrane receptors composed of α- and β-subunits, are also indispensable for appropriate locomotion (18). As cell-matrix adhesion proteins, they are integral for the highly coordinated cell migration cycle involving the polarization of the leading edge, adhesion to the extracellular matrix (ECM), extension and translocation of the cellular body, and lastly detachment at the cellular rear by contraction of the actin cytoskeleton (19, 20, 21). Upon binding to the ECM, integrins however not only coordinate cell-matrix-adhesion but are also involved in the polymerization of the actin cytoskeleton at the leading edge allowing correct cell polarization. This is facilitated by its link to the cytoskeleton by actin-binding proteins like vinculin or by its downstream modulators, focal adhesion kinases and Src-kinases (10, 18, 22, 23). Targeting the cell-ECM interface is thus of great interest for the development of new antimetastatic therapeutics and integrin-targeting antibodies or drugs have been repeatedly investigated in clinical trials (24, 25).

To fulfil their function, integrins have to be continuously recycled from and to the leading edges of migrating cells to enable a spatiotemporal restriction of focal adhesion sites required for the extension of the cellular body (26, 27). Accordingly, E-cadherin is maintained and modulated at the plasma membrane by the internal trafficking machinery facilitating a precise regulation of cell-junctional integrity (28, 29). Taken together, this suggests the targeting of intracellular trafficking as a suitable antimigratory and therefore antimetastatic strategy, as it targets proteins responsible for both cell–cell and cell-matrix contacts. In this context, lysosomal membrane proteins are in the focus of intense research as crucial regulators of endocytosis, intracellular transport, and exocytosis (30). Recently endolysosomal cation channels have emerged as an attractive anticancer target: namely the mucolipin subfamily of transient potential receptors (TRPMLs), which comprises three isoforms—TRPML1 (MCOLN1), TRPML2 (MCOLN2), and TRPML3 (MCOLN3) (31). TRPML1, the most intensively researched member of the family, is ubiquitously expressed in the membranes of endosomes and lysosomes, whereas TRPML2 and TRPML3 are mainly localized in specialized cells (*e.g.,* immune cells, hair cells of the inner ear, secretory cells, and melanocytes) (32, 33). In past research TRPML1 has been linked to ion homeostasis, vesicular trafficking, and autophagy (34, 35). Aside from these physiological functionalities, TRPML1’s role in cancer is emerging. Interestingly, it has been implicated to regulate cancer cell migration as its inhibition reduces invasiveness of breast cancer cells *in vitro* (34) and *in vivo* (36). However, the underlying mechanism is still vastly elusive. Given the apparent correlation between TRPML1 and cancer cell migration, we aimed to further elucidate its role in cancer cell migration and, most importantly, uncover the underlying mechanisms by monitoring cell-junctional and cell-adhesion proteins.

## Results

### TRPML1 loss of function reduces cancer cell migration and adhesion

For this study, we chose a panel of different cancer cell lines, including human and murine cell lines from hepatocellular carcinoma (RIL-175, Huh-7, HepG2, Hep3B), melanoma (SkMel-5, B16F10-luc), and breast cancer (MDA-MB-231, SkBr-3). To further study the impact of loss of TRPML1 on migration, we worked with TRPML1 KO clones (RIL-175, SkMel-5, B16F10-luc, MDA-MB-231) or transient siRNA knockdown (KD) cells (Huh-7, HepG2, Hep3B, SkBr-3) (Fig. S1*B*). For MDA-MB-231 and SkMel-5 cells, TRPML1 KO has previously been established (34). For RIL-175 and B16F10-luc (Fig. S1, *E* and *F*) cells we performed a KO of TRPML1 using the CRISPR/Cas9 system, as previously reported (32, 34).

**Figure 1:**
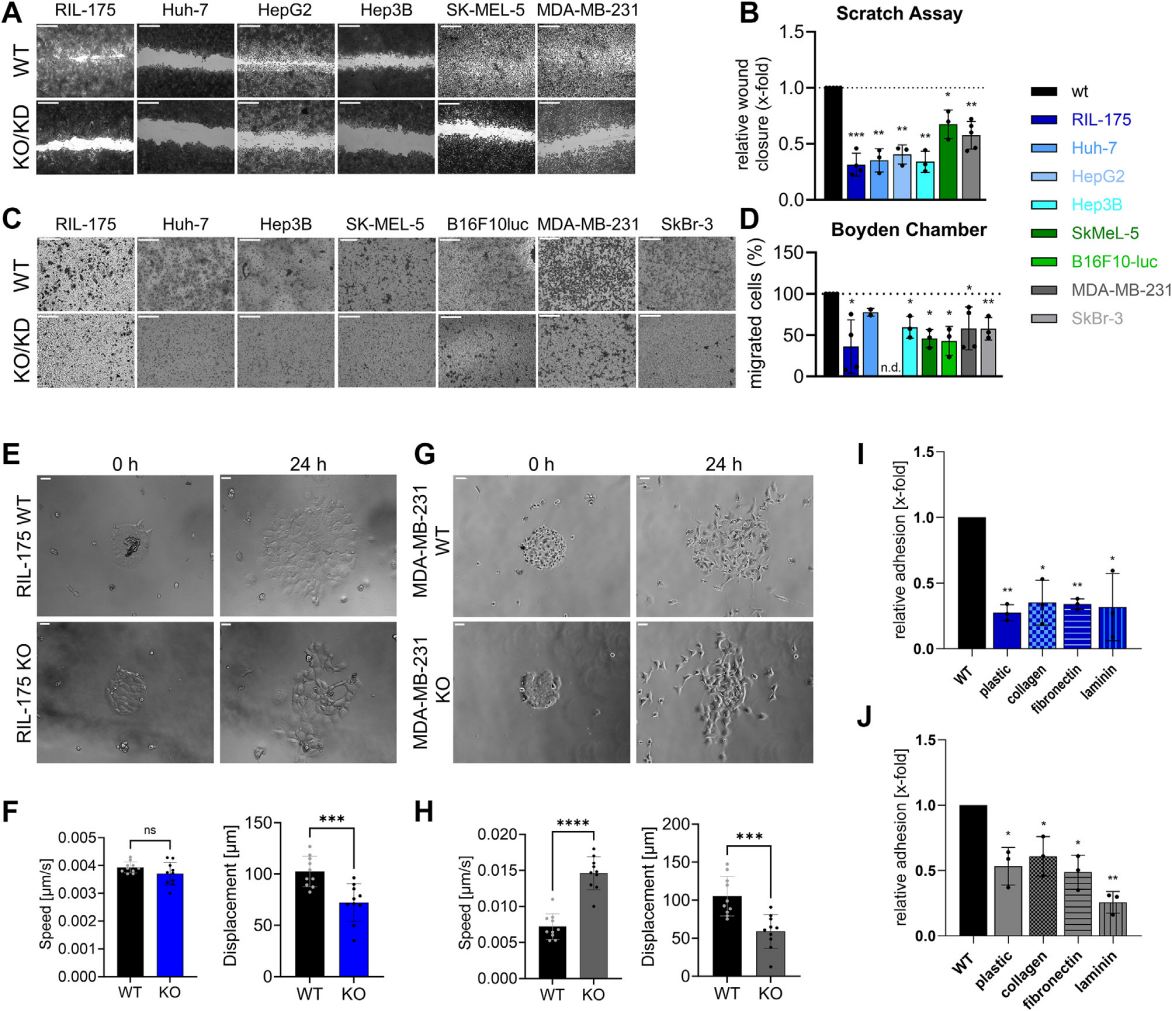
**TRPML1 loss of function reduces cancer cell migration and adhesion.***A* and *C,* images are representative. The scale bars represent 20 μm (*A*), wound-healing, and (*C*) Boyden-Chamber experiments along FCS gradient show fixed and *crystal-violet* stained cells after migration for 6 h (RIL-175, B16F10-luc), 20 h (Huh-7, HepG2, Hep3B) or 24 h (MDA-MB-231). *B* and *D,* significantly decreased migration for KO/KD cells is depicted by quantification. *E*–*H*, speed and displacement analysis of micropatterning experiments. The scale bars represent 100 μm. *I* and *J,* quantification of adhesion of WT and KO cells seeded onto substrates as indicated for RIL-175 (*I*) and MDA-MB-231 (*J*). Statistical significance was assessed by unpaired student’s *t* test with Welch’s correction ∗*p* < 0.0332, ∗∗*p* < 0.0021, ∗∗∗*p* < 0.0002 and ∗∗∗∗*p* < 0.001. FCS, fetal calf serum; KD, knockdown; ns, not significant.

We analyzed migration behavior of KO/KD cells compared to their parental lines in different *in vitro* migration assays. Horizontal 2D cell migration in a wound-healing assay and migration in a Boyden-Chamber were significantly reduced upon KO/KD of TRPML1 (Fig. 1, *A*–*D*). Furthermore, live-cell imaging data allowed us to monitor collective cell migration and exclude reduced proliferation behavior of the RIL-175 KO cells to be responsible for reduced migration (Video S1). Consistently, live-cell imaging of a micropatterned platform allowing the time-controlled cell migration outside of a highly cell-adhesive fibronectin ring further revealed that the KO of TRPML1 predominantly reduced the displacement of the cells (Fig. 1, *E*–*H*).

In addition, we used an *“in-vivo like”* 3D spheroid system to corroborate the antimigratory effect of TRPML1 inhibition. We could show that the spheroid diameters and area in RIL-175 cells were reduced significantly upon treatment with selective TRPML1-inhibitor estradiol methyl ether (EDME) (34) (Fig. S1*C*). Furthermore, we performed an adhesion assay by seeding the cells onto different coating conditions representing some of the proteins that make up the macromolecular network of the ECM, including collagen, fibronectin, and laminin (37). Indeed, RIL-175 and MDA-MB-231 KO cell lines displayed reduced adhesion to all employed ECM ligands, as analyzed by confocal microscopy (Figs. 1, *I* and *J* and S2). Taken together, these findings clearly depict that the reduction/loss of TRPML1 inhibits cancer migration in different kinds of cancer in 2D and 3D systems and its adhesion properties.

### Loss of TRPML1 function reduces dissemination of cancer cells *in vivo*

**Figure 2:**
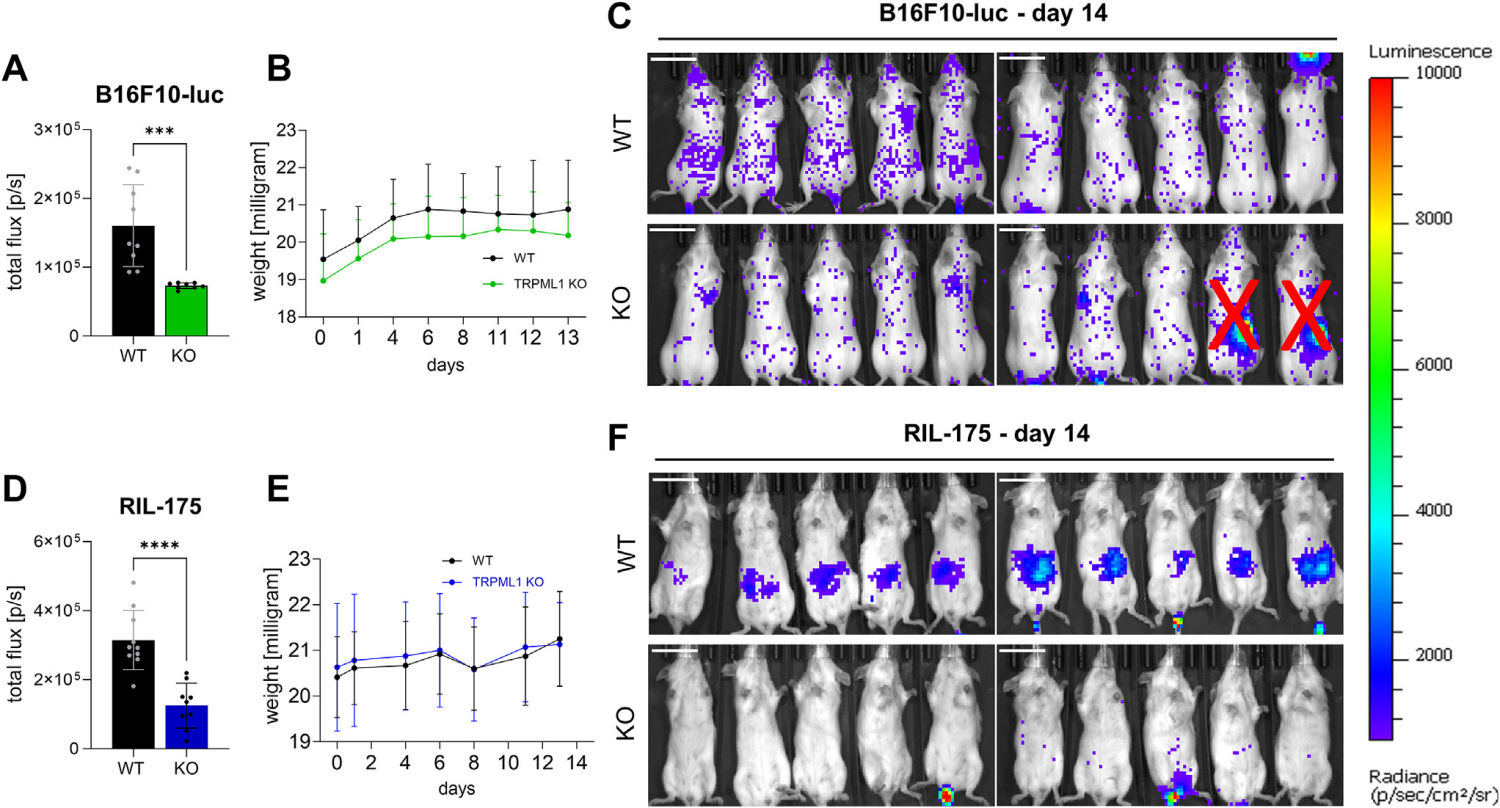
**TRPML1 KO reduces dissemination of tumor cells *in vivo*.***A,* total flux on day 13 (endpoint). *B,* weights of all mice was documented over the whole experimental period. *C,* images of mice receiving 200,000 B16F10-luc WT or TRPML1 KO cells (day 0) after intraperitoneal injection of 6 mg/ml luciferin on day 13. Images show the whole group. Two mice were identified as outliers *via* ROUT test, Q = 1% and excluded from evaluation. The scale bar represents 2 cm. *D,* total flux on day 13 (endpoint). *E,* weights of all mice was documented over the whole experimental period. *F,* images of mice receiving 200,000 RIL-175 WT or TRPML1 KO cells (day 0) on day 13. The scale bar represents 2 cm. Statistical significance was assessed by unpaired student’s *t* test ∗∗∗*p* < 0.0002 and ∗∗∗∗*p* < 0.001.

To examine tumor invasiveness and dissemination potential, we chose an *in vivo* tumor model with murine melanoma (B16F10-luc) cells and murine hepatocellular carcinoma (RIL-175) cells. To this end, we first generated murine TRPML1 CRISPR/Cas9 KOs in B16F10-luc cells (Fig. S1, *E* and *F*). Then, C57BL/6BrdCrHsd-Tyrc mice were injected with TRPML1 KO RIL-175, or TRPML1 KO B16F10-luc, or the respective WT cells. In line with our *in vitro* data, both groups injected with TRPML1 KO cells displayed reduced overall dissemination compared to the respective WT group (Fig. 2, *C* and *E*). Furthermore, we note that RIL-175 WT mice displayed elevated tumor dissemination in the liver compared to TRPML1 KO mice (Fig. 2, *D* and *E*).

These data corroborate our hypothesis that loss of TRPML1 function reduces invasive capacities and the dissemination of tumor cells *in vivo*.

### Loss of TRPML1 function disrupts actin polymerization and reduces cytosolic calcium levels

**Figure 3:**
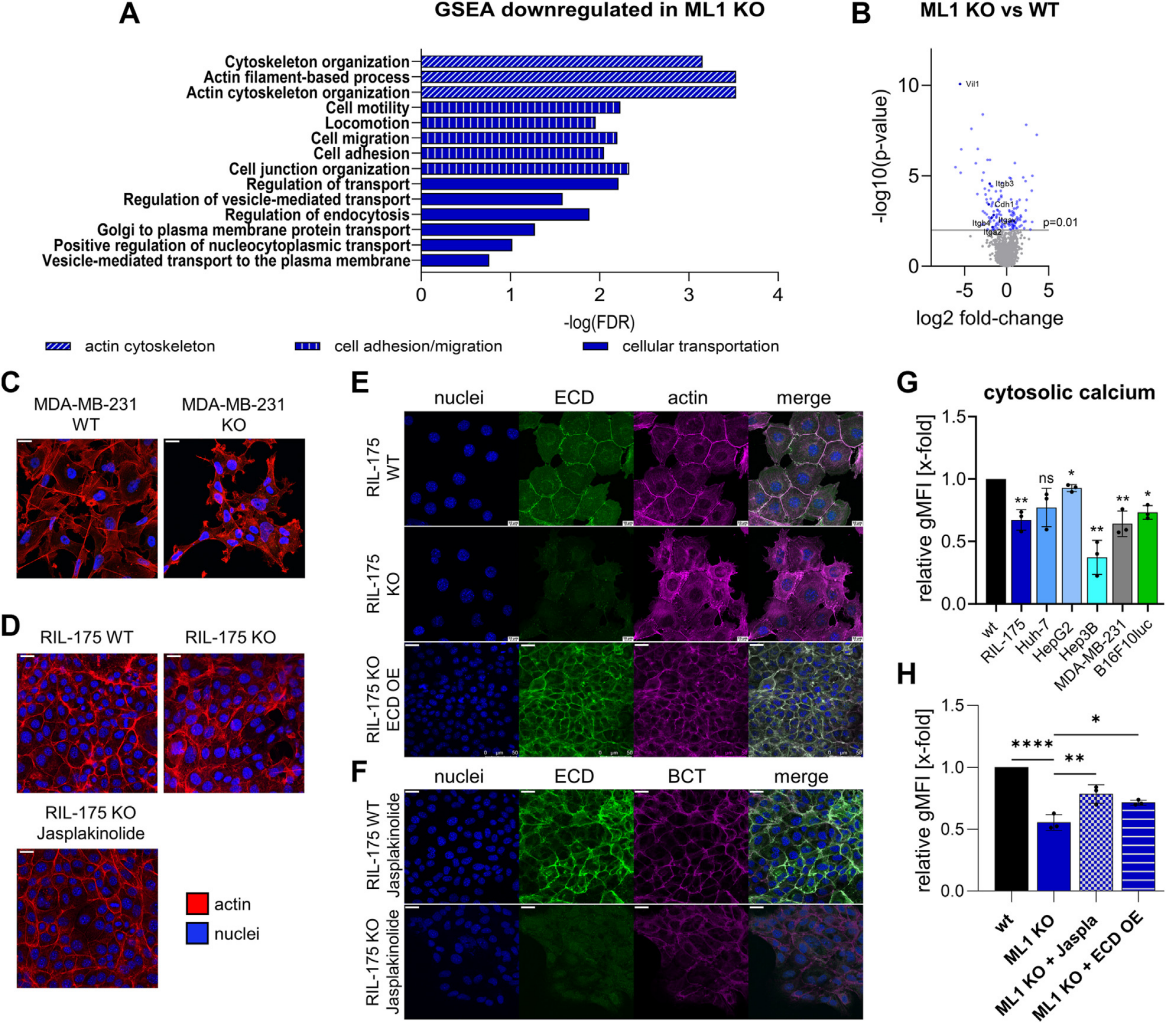
**The ablation of TRPML1 function reduces calcium levels and disrupts actin polymerization.***A,* gene set enrichment analysis (GSEA) revealed significantly enriched gene sets for RIL-175 KO cells in comparison to RIL-175 WT cells with an false discovery rate q-value ≤ 0.05. Proteomic analysis was performed by LC-MS/MS. The *x*-axis represents the enrichment significance in –log10(FDR *q*-value). *B, volcano plot* of the proteomic data significantly altered proteins is highlighted in *blue*. *Dark blue* data points are being discussed in the following. *C* and *D,* MDA-MD-231 and RIL-175 WT and KO cells stained for actin and nuclei. Representative images were shown. Experiment was performed n = 3 (RIL-175) and n = 2 (MDA-MD-231). RIL-175 KO cells were also pretreated with 100 nM Jasplakinolide for 24 h (*D*). The scale bar represents 20 μm. *E,* RIL-175 WT, KO, and E-cadherin–overexpressing cells were stained for E-cadherin, actin, and nuclei. *F,* RIL-175 WT and KO cells were stimulated with 100 nM Jasplakinolide for 24 h and then stained for E-cadherin, β-catenin, and nuclei. The scale bar represents 20 μm. *G* and *H*, cytosolic calcium levels were assessed using calcium-520 AM dye, detection through flow cytometry, and determination of relative gMFI levels. Statistical significance was assessed by unpaired student’s *t* test with Welch’s correction ∗*p* < 0.0332, ∗∗*p* < 0.0021 and ∗∗∗∗*p* < 0.001. E-cadherin, epithelial-cadherin; ns, not significant.

To further elucidate the underlying mechanism for the observed antimigratory and antiadhesive effects *in vitro* and *in vivo*, we conducted an unbiased proteome analysis in RIL-175 WT and KO cells, as recently described by our group, in which a total of 1968 proteins were identified (32). A functional gene set enrichment analysis (GSEA) between WT and KO cells confirmed downregulation of pathways required for appropriate migration, including cell adhesion, locomotion, as well as cytoskeleton organization upon KO of TRPML1 (Fig. 3*A*). We found E-cadherin, β-integrin, and Villin-1 to contribute to many downregulated biological process pathways (Figs. 3*B* and S3*A*). Thus, we focused on these three protein groups responsible for the downregulated pathways GO Terms.

Villin-1 is a Ca^2+^-regulated actin-modifying protein also responsible for actin nucleation capping and bundling of actin filaments (38). Indeed, we could observe altered actin polymerisation in RIL-175 and MDA-MB-231 KO cell lines (Fig. 3, *C* and *D*). In this context, we checked for cytosolic calcium levels and could observe significant downregulation upon KO/KD of TRPML1 (Fig. 3*G*). Alongside, confocal images revealed that the loss of TRPML1 function reduces E-cadherin levels at membrane site and therefore colocalization with actin filaments (Fig. 3*E*). While actin filament structures (Fig. 3*D*) and cytosolic calcium levels (Fig. 3*H*) could partially be rescued by adding actin polymerization agent Jasplakinolide, E-cadherin levels remained unaltered (Fig. 3*F*). Reexpression of E-cadherin levels could only be achieved through transfection with an E-cadherin–overexpressing vector (Fig. 3*E*).

Our results indicate that the reduction of intracellular calcium might be regulating the depolymerization of actin filaments in KO cell, and thereby migratory events, as elevating calcium levels with Jasplakinolide could rescue the WT phenotype. Furthermore, the loss of polymerized actin is accompanied by destabilized adherens junctions (AJ) complexes at cell–cell and cell-matrix contact site and therefore reduced adhesive traits (Figs. 1, *I* and *J* and S3*B*).

### Knockout of TRPML1 affects cell adhesion by abrogating β_1_-integrin receptor trafficking

In addition to reduced cytoskeleton organization, GSEA revealed reduced cell adhesion upon KO of TPRML1 (Fig. 3*A*) and the downregulation of significant cell-matrix proteins *Itgav, Itga2, Itgb4, Itgb3* (Figs. 3*B* and S3*A*).

**Figure 4:**
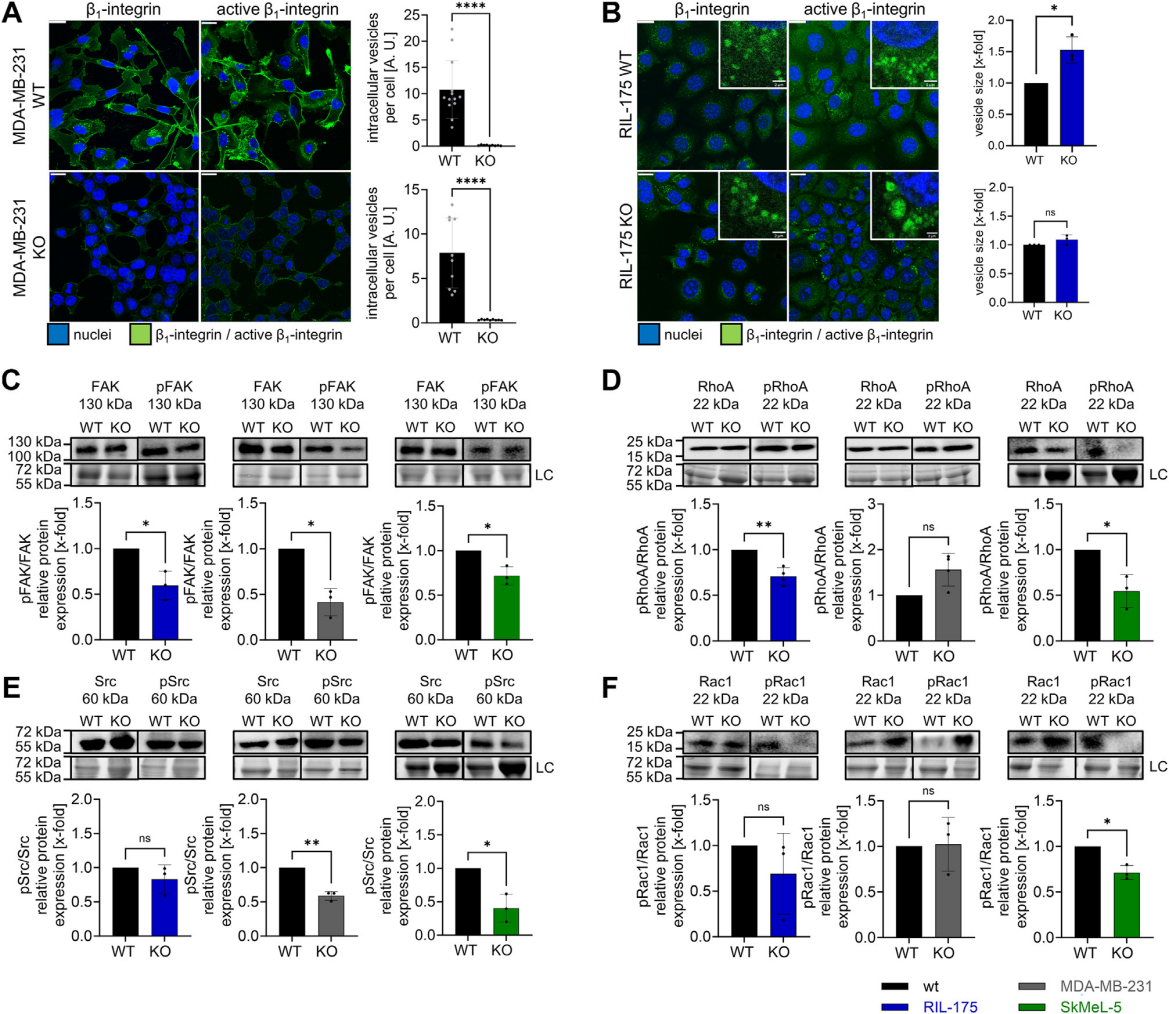
**The KO of TRPML1 affects cell adhesion by abrogating β**_**1**_**-integrin receptor trafficking.***A* an*d B,* MDA-MB-231 and RIL-175 WT and KO cells stained for β_1_-integrin and active β_1_-integrin and the nuclei. The vesicle size was quantified by ImageJ and normalized to the WT level. *C–F,* relative protein levels of FAK and pFAK, Src and pSrc, RhoA and pRhoA, Rac1 and pRac1. Images/blots are representative. The scale bars represent 20 μm or 2 μm (zoom-ins). For MDA-MB-231 WT and KO cells, Src and Rac1 were blotted on the same membrane. Conveniently for SkMel-5 RhoA and Src blotting the same membrane was used due to different protein sizes. Loading control = LC. Statistical significance was assessed by unpaired student’s *t* test. ∗*p* < 0.0332 and ∗∗*p* < 0.0021. ns, not significant.

Cell-matrix adhesion is predominantly mediated by the family of integrins (23). They undergo constant recycling processes at the leading edge of migrating cells allowing a spatiotemporal restriction of adhesive sites (26, 27). Despite attenuated cell-matrix adhesion, β_1_-integrin-activity itself remained unaltered in both KO cell lines (Fig. S3*E*). However, confocal microscopy revealed a diffusely accumulated β_1_-integrin-receptor (total and active form) at the plasma membrane of MDA-MB-231 KO cells (Fig. 4*A*) contrasting the distinctive integrin-containing vesicles in the cytoplasm of the parental cell line. Disturbed endocytosis was confirmed by a receptor-internalization assay showing that only the WT cells could properly internalize the β_1_-integrin receptor (Fig. S3*G*). In contrast, unaltered β_1_-integrin internalization was detected in RIL-175 KO cells (Fig. 4*B*), indicating unaffected endocytosis. Consistently, enlarged β_1_-integrin vesicles (Fig. 4*B*) implicated trapping of the receptor in endolysosomal vesicles corroborating hampered recycling processes in RIL-175 KO cells. Additionally, dysfunctional β_1_-integrin trafficking is reflected in altered integrin downstream signaling. Loss of TRPML1 function results in downregulated focal adhesion kinase activity in three KO cell lines and reduced Src-activity in SkMel-5 and MDA-MB-231 KO cells (Fig. 4, *C* and *E*). RIL-175 and SkMel-5 KO cells additionally displayed lowered RhoA-activity (Fig. 4*D*). Despite unaltered Rac1 protein levels (Fig. 4*F*), Rac1 appears to be less condensed at the cellular front (Fig. S3, *C* and *D*). Rac1 is indispensable for actin polymerization at protrusion site (20, 39), consequently lamellipodia formation is hampered (Fig. S3, *C* and *D*). Taken together, the data suggest that disturbed β_1_-integrin receptor recycling upon KO results in downregulation of β_1_-integrin signaling cascade downstream, which then is reflected in the abatement of distinctive migratory and adhesive properties.

### Impaired TRPML1 function hampers AJ integrity

**Figure 5:**
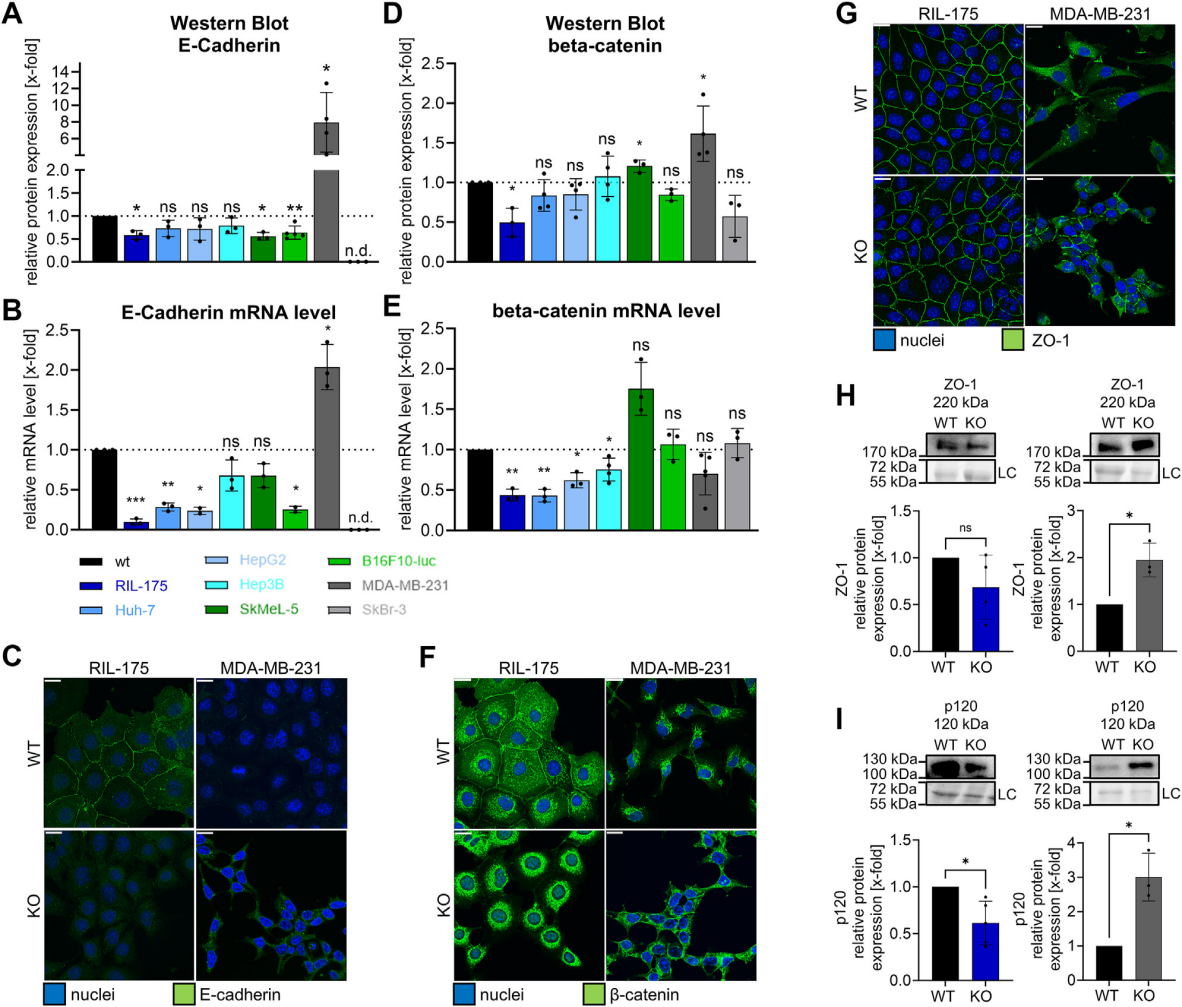
**Disrupted adherence junction upon KO of TRPML1.***A*, *D*, *H*, and *I,* relative protein levels of E-cadherin (*A*), β-catenin (*D*), ZO-1 (*G*), and p120 (H). Images/blots are representative. Loading control = LC. *B* and *E*,) relative mRNA levels E-cadherin (*B*) and β-catenin (*E*) acquired by RT-qPCR. *C*, *F*, and *G,* representative confocal images showing E-cadherin (*C*), β-catenin (*F*), and ZO-1 (*G*) and the nuclei. The scale bar represents 20 μm. Statistical significance was assessed by unpaired student’s *t* test with Welch’s correction ∗*p* < 0.0332, ∗∗*p* < 0.0021, and ∗∗∗*p* < 0.0002. E-cadherin, epithelial-cadherin; RT-qPCR, real-time quantitative PCR; ns, not significant.

To further investigate the observed downregulation of E-cadherin, we first checked protein and mRNA levels of E-cadherin in our panel of cell lines. Interestingly, E-cadherin protein levels were significantly downregulated in all KO cell lines, except MDA-MB-231 KO cells (Fig. 5*A*). In line, inhibiting the channel with EDME (50 μM) significantly decreased E-cadherin protein expression in RIL-175 cells (Fig. S4*D*). The contrasting upregulation of E-cadherin level in MDA-MB-231 KO cells could also be reflected in mRNA levels and confocal images (Fig. 5, *D* and *G*). Next, we checked, whether a downregulation of E-cadherin might influence associated AJ complex proteins, for example, β-catenin. For E-cadherin–associated proteins β-catenin and p120, we observed decreased protein levels in RIL-175 KO cells and vice versa in MDA-MB-231 KO cells (Fig. 5, *B*, *E*, and *I*). Accordingly, β- and p120-catenin were condensed at the plasma membrane of MDA-MB-231 KO cells, whereas removal of those proteins was evident for RIL-175 KO (Figs. 5*H* and S4*C*). In line, E-cadherin and β-catenin were colocalized at the plasma membrane in WT cells but not in KO cells (Fig. S3*B*).

Secondly, we found protein expression of tight-junction–associated protein ZO-1 (40) not to be altered in RIL-175 KO cells. In contrast, MDA-MB-231 KO cells displayed an increase in ZO-1 protein and consequently more tight-junctional contacts between neighboring cells (Fig. 5*C*).

Of note, both KO cell lines expressed significantly increased N-cadherin protein levels (Fig. S4, *E* and *F*) despite MDA-MB-231 KO cells displaying elevated E-cadherin. The loss of E-cadherin and consequent increase in N-cadherin is one of the hallmarks of the EMT (9), excluding EMT as possible mechanism in MDA-MB-231 cells.

In total, the data suggests TRPML1 as a key regulator to recruit AJ complex components E-cadherin and β-catenin to the cell membrane and therefore secure promigratory at cell–cell or cell-matrix contact site (14). Furthermore, we conclude that a full loss of function is required to properly describe the phenotype upon reduction of TRPML1 function, as KD cells displayed a nonsignificant reduction of both AJ components. Thus, in the following, we focus on the two alleged opponents RIL-175 and MDA-MB-231 cells to further investigate the intriguing underlying cellular mechanism.

### Knockout of TRPML1 impedes intracellular trafficking

E-cadherin levels can be altered by various modulators. For instance, endocytosis-exocytosis processes have emerged as strong E-cadherin modulators (28, 29). The performed GSEA revealed gene sets for endocytosis, intracellular transport, and exocytosis are downregulated in RIL-175 KO cells (Fig. 3*A*).

In this context, vesicle tethering, that is the building of protein complexes that physically connect a transport vesicle to its target membrane prior to fusion (41), a crucial step in trafficking processes, is mediated by Rab-proteins (42). Internalized proteins (*e. g.*, E-cadherin) are transferred from endocytic vesicles to Rab5-positive early endosomes (EEs) and can be rerouted from recycling endosomes to the cell membrane *via* Rab11 (29).

**Figure 6:**
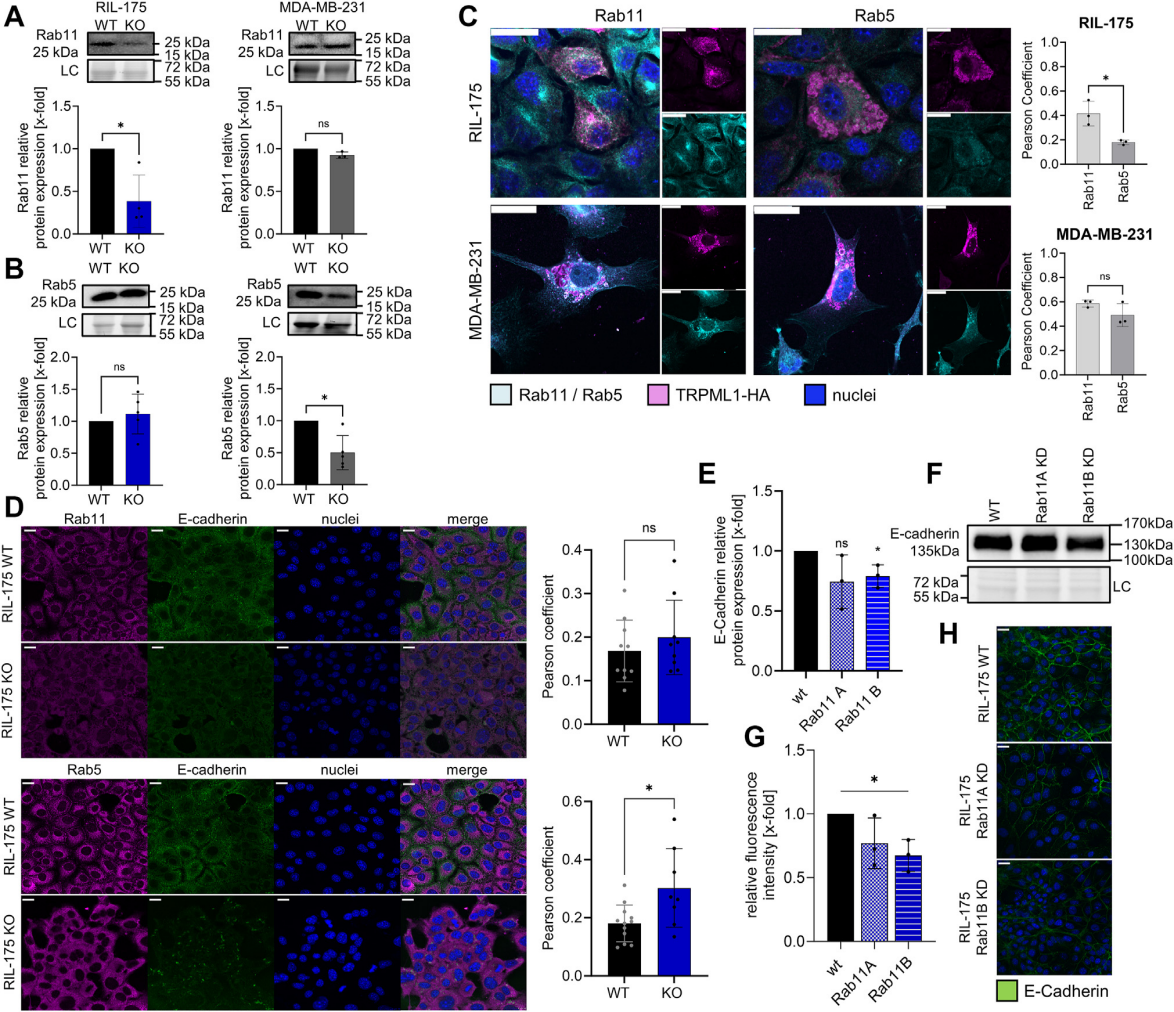
**TRPML1 and Rab11/Rab5.***A* and *B,* relative protein levels of Rab11 and Rab5 in RIL-175 and MDA-MB-231 WT and KO cells. Loading control = LC. *C,* colocalization of TRPML1 (*magenta*) with Rab5 or Rab11 (*cyan*). The nucleus is shown in *blue* (Hoechst). *D,* RIL-175 cells were stained for Rab11 or Rab5, E-cadherin, and nuclei. *C* and *D,* quantification of the colocalization as analyzed by ImageJ. *E* and *F,* relative protein level of E-cadherin after Rab11A/B KD. *H,* confocal images showing E-cadherin and nuclei of RIL-175 WT cells after Rab11A/B KD and the associated quantification (*G*). Images/Blots are representative. The scale bars represent 20 μm. Quantification of fluorescence intensity was assessed with ImageJ. Statistical significance was assessed by unpaired student’s *t* test with Welch’s correction. ∗*p* < 0.0332. E-cadherin, epithelial-cadherin; KD, knockdown; ns, not significant.

We checked Rab11 and Rab5 levels in both cell lines and observed significant reduction of Rab11 in RIL-175 KO cells on mRNA level (Fig. S5*G*) and protein level (Fig. 6*A*), corroborating a predominant effect on the exocytotic machinery. This finding is opposed to the unaffected protein levels of Rab5 in RIL-175 KO cells, indicating that TRPML1 has no effect on the level of Rab5-positive EEs endocytosis (Fig. 6*B*).

In contrast, Rab5 was significantly reduced in MDA-MB-231 KO cells (Fig. 6*B*) highlighting aberrant trafficking after endocytosis. Concurrently, a TRPML1-HA-tag fusion protein colocalizes with Rab5 and Rab11 in both WT cell lines. We were not able to observe any significant difference in the colocalization of TRPML1 with Rab5 or Rab11 in MDA-MB-231 cells (Fig. 6*C*). By contrast, RIL-175 cells showed a significantly larger colocalization of TRPML1 with Rab11 than with Rab5 (Fig. 6*C*). Likewise, we could observe an increased colocalization of E-cadherin and Rab5 upon KO, whereas colocalization of E-cadherin with Rab11 remained unaltered (Fig. 6, *D* and *E*). A transient KD of Rab11B resulted in a reduction of whole protein levels of E-cadherin (Fig. 6*F*) and its expression at membrane site (Fig. 6, *G* and *H*).

In this context, Rab11, a prominent marker of recycling endosomes, has emerged as an important regulator of E-cadherin (43, 44). After transient Rab11 overexpression in RIL-175 TRPML1 KO cells, we observed an increase in E-cadherin on protein level (Fig. S5*B*). Consistently, E-cadherin was effectively reintroduced at the plasma membrane (Fig. S5*F*).

Proteins cannot solely be recycled *via* Rab11 route but also in a fast-recycling track occurring directly from EEs (28, 29). In RIL-175 KO cells, this pathway is unaffected, as shown by the unaltered recovery of an E-cadherin GFP-fusion protein in fluorescence recovery after photobleaching (FRAP) experiments (Fig. S5, *C* and *D*). Lastly, we observed no effect of blocking lysosomal degradation with chloroquine on E-cadherin protein expression, indicating no involvement of disrupted lysosomal function in the observed mode of action (Fig. S5*E*).

**Figure 7:**
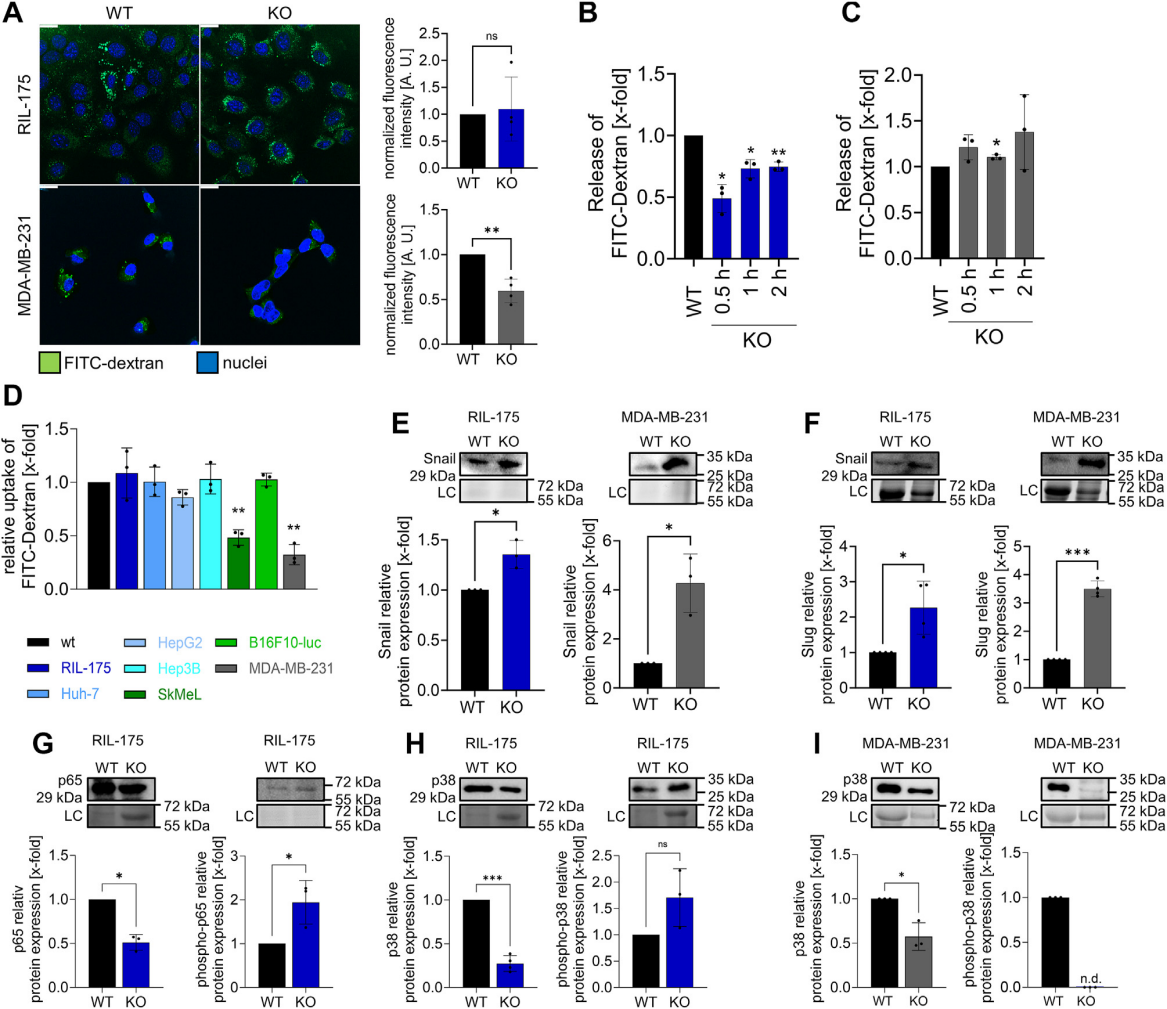
**TRPML1 KO impedes intracellular trafficking.***A,* internalized FITC dextran (200 μg/ml, incubated for 2 h) and the nucleus nuclei. Fluorescence intensities were measured by ImageJ and normalized to the number of cells per image. *B* and *C,* lysosomal exocytosis assay of released FITC dextran (200 μg/ml, incubated for 24 h) upon calcium treatment (50 mM). The results were normalized to the WT level. *D,* flow cytometry analysis of endocytosed FITC dextran (200 μg/ml) after an incubation time of 2 h. *E–I,* relative protein levels of snail (*E*), slug (*F*), p65/pp65 (*G*), p38/pp38 (*H* and *I*) in RIL-175, and MDA-MB-231 WT and KO cells. Images/blots are representative. Loading control = LC. The scale bars represent 20 μm. Quantification of fluorescence intensity was assessed with ImageJ. Statistical significance was assessed by unpaired student’s *t* test with Welch’s correction. ∗*p* < 0.0332, ∗∗*p* < 0.0021, and ∗∗∗*p* < 0.0002. ns = not significant.

Furthermore, we investigated cellular uptake in RIL-175 and MDA-MB-231 cells with the use of FITC-dextran. In line, uptake in RIL-175 cells remains unaltered upon TRPML1 KO (Fig. 7*A*), and we observed a reduction of exocytotic activity in RIL-175 KO cells indicated by a diminished release of β-hexosaminidase (Fig. S6*B*) and FITC-dextran (Fig. 7*B*).

In contrast, for MDA-MB-231 KO cells, we noted a largely reduced endocytic uptake of FITC-dextran (Fig. 7*A*), whereas exocytosis remained unaltered (Fig. 7*C*).

Supporting these observations, we observed that after triggering exocytosis with ionomycin, MDA-MB-231 KO cells displayed significantly reduced cathepsin B release into the medium, whereas no effect was observable for RIL-175 KO cells (Fig. S6, *D* and *E*). Cancer cells often release the lysosomal protease cathepsin B for collagen degradation in the ECM by vesicular exocytosis (45).

Hence, taken together, the data strongly supports a pivotal and differential role of TRPML1 on Rab5 and Rab11 on the recycling of E-cadherin. While endocytic uptake is evidently hampered in MDA-MB-231 KO cells, RIL-175 KO cells display abrogated exocytosis.

### E-cadherin/NF-κB feedback loop regulating E-cadherin expression

Finally, our results indicate that E-cadherin is also regulated in a transcriptional manner, as mRNA levels alter differently upon loss of KO (Fig. 5, *D* and *E*). Past studies highlight that the loss of E-cadherin levels promote the activation of tumor suppressor NF-κB and cytoplasmic β-catenin (46, 47). Our results show that the loss of E-cadherin is accompanied by a reduction of colocalization with β-catenin, whilst β-catenin levels remain mostly unaltered (Fig. 5*B*). Therefore, we checked for protein levels of NF-κB factor p65 and p38 in RIL-175 and MDA-MB-231. In fact, we observed a significant downregulation of p38 or p65 and subsequently an upregulation of phosphorylated p38 and p65 protein levels in RIL-175 (Fig. 7, *G* and *H*). In contrary to RIL-175, but line with previous observations MDA-MB-231 cells show no expression of phosphorylated-p38 (Fig. 7*I*) and therefore possibly no p38-mediated NF-κB phosphorylation. The observed p38-mediated NF-κB activation is known to suppress E-cadherin expression by promoting the expression of E-cadherin suppressors, for example, snail, slug, and ZEB1/2 (48, 49, 50, 51, 52). Interestingly, snail and slug levels were not only elevated in RIL-175 cells but also in MDA-MB-231, despite upregulation of E-cadherin expression (Fig. 7, *E* and *F*). Thus, we conclude that the reduction (RIL-175) or the elevation (MDA-MB-231) of E-cadherin levels at membrane site regulated by impaired trafficking and disrupted actin polymerization upon loss of TRPML1 function, leads to a p38-mediated activation (RIL-175) or inhibition (MDA-MB-231) of NF-κB and therefore form transcriptional feedback in the expression of E-cadherin.

## Discussion

This study designates TRPML1 as a promising target for the treatment of invasive cancers, as KO/KD of TRPML1 resulted in loss of actin morphology and the reduction of adhesive and migratory capacities (Fig. 1, *A* and *B*).

One must mention that TRPML1 is suggested to be the main lysosomal Ca^2+^ cation channel, which has shown to influence not only lysosomal pH but also intracellular Ca^2+^ concentrations through lysosomal nanodomains (53, 54, 55). The second messenger Ca^2+^ influences, that is, vesicle fusion and fission (56, 57, 58) and Rab proteins (59, 60, 61, 62). In line with the literature, we could observe a significant reduction of intracellular calcium upon loss of TRPML1 function (Fig. 3*G*). Previous research has highlighted the importance of TRPML1-mediated Ca^2+^-flux in cellular trafficking (63), lysosomal exocytosis (64), and in interaction with fusion proteins in a Ca^2+^-dependent manner (65, 66). In our study, proteome analysis and the following GSEA revealed Villin-1 to be significantly downregulated upon loss of TRPML1 function. Villin-1 is a Ca^2+^-dependant actin-modifying protein, which is involved in the nucleation, capping, and bundling of actin filaments. Indeed, we could observe altered actin polymerization in KO cells, and were able to restore actin structures by stimulation with actin polymerization agent Jasplakinolide, which was linked with rescued calcium levels.

That in mind, KO cells displayed less condensed Rac1 at the leading edge, consequently hampered lamellipodia formation (Fig. S3, *C* and *D*). Rac1 is key regulator for cytoskeleton organization, including actin polymerization and lamellipodia formation. It has been shown that Rac1 activation and translocation is regulated by intracellular calcium levels (67). Price *et al.* showed that elevated intracellular calcium concentrations induced activation of PKC, which in return phosphorylates RhoGDI alpha and induce translocation of cytosolic Rac to membrane sites (67). Furthermore, Vestre *et al*. linked Ca^2+^ release from TRPML1 to the activation of myosin phosphorylation, thereby triggering localized actomyosin contractility (68). In line, their KO of TRPML1 was associated with alterations in actin distribution and therefore altered migration behavior of dentritic cells (68). Taken together, we propose TRPML1-mediated Ca^2+^ flux to be a suitable mode of action for the reduction of Rac1 activation at the leading edges, alterations in actin polymerization and consequently in altered migration behavior.

Concurrently, we observed altered β_1_-integrin signaling due to aberrant β_1_-integrin receptor internalization (MDA-MB-231) and recycling (RIL-175) (Fig. 4*A*), which consequently manifested in reduced adhesive properties of the KO cell lines (Fig. 1, *I* and *J*). Focal adhesion site formation at leading edges and in cell–cell contact sites is required for efficient locomotion, which is predominantly mediated by the intracellular trafficking machinery (27, 69). Attenuated β_1_-integrin recycling has already been reported to impair cell adhesion and migration by the inhibition of two-pore channel 2, another endolysosomal calcium channel (70), which is likely attributed to downregulated Ca^2+^-signaling upon two-pore channel 2 inhibition. Furthermore, TRPML1 regulates endolysosomal fusion in Drosophila (63) by modifying intracellular Ca^2+^-levels, as previously postulated (70). TRPML1 also regulates Ca^2+^-dependent lysosomal exocytosis (64) and has been implicated to interact with fusion proteins in a Ca^2+^-dependent manner (65, 66) proposing altered trafficking upon loss of TRPML1 function a suitable mode of action for our findings.

In the context of loss of adhesive properties, loss of TRPML1 function led to a reduced colocalization of AJ complex molecules E-cadherin and β-catenin, simultaneously E-cadherin diminished from plasma membrane in RIL-175 cells (Fig. S3*B*). Despite markedly reduced migration, we observed downregulation of E-cadherin in RIL-175 KO cells (Fig. 1). At first glance, this is counterintuitive as E-cadherin is a widely established tumor suppressor (71, 72). Loss of E-cadherin is typically associated with highly invasive tumors, upon which cells gain a mesenchymal phenotype and can individually invade the surrounding tissue due to the loss of cell–cell adhesion (15). Intriguingly, as shown by Haraguchi *et al*., the loss of E-cadherin impedes promigratory RhoA- and Rac1-signaling, and thereby reduces the migratory capacity of E-cadherin KO RMG-1 cells (73). Consistently, E-cadherin is a crucial regulator of actin dynamics and accordingly the lamellipodia and filopodia formation due to its direct link to the actin cytoskeleton via β- and α-catenin (10). Indeed, we could not only observe hampered RhoA-activity (Fig. 4*D*) but also as mentioned delocalized Rac1 at the leading edge and reduced colocalization of E-cadherin with actin and β-catenin. Moreover, the loss of adhesive properties in RIL-175 cells can be described by the reduction of total cytosolic Ca^2+^, as Ca^2+^ is needed to secure rigidness of E-cadherin to bind β- and α-catenin at cell membrane site (74).

In contrary, in mesenchymal MDA-MB-231 WT cells an elevation of E-cadherin levels upon loss of TRPML1 function was observed (75) (Fig. 5). This elevation was associated with a reestablishment of the epithelial phenotype in KO cells. Our observations are in line with the findings of Merk *et al*., who have shown that impairing lysosomal function by V-ATPase inhibition resulted in impaired E-cadherin internalization and increased E-cadherin surface levels alongside reduced migration in a breast cancer cell model (76). Other research also highlighted the possibility of hampering cancer cell migration by increasing E-cadherin, further corroborating its status as tumor suppressor (14). Accordingly, Chao *et al*. and Mbalaviele *et al*. observed reduced migratory capacities in MDA-MB-231 cells after stable introduction of E-cadherin both *in vitro* and *in vivo* (75, 77).

Of note, MDA-MB-231 and SkBR-3 are naturally E-cadherin negative cells compared to all other tested cell lines, as *Cdh1* expression is suppressed in WT cells by methylation of the promotor region (78, 79). The significant upregulation of E-cadherin mRNA levels in MDA-MB-231 cells and the contrary significant downregulation in all other KO cell lines, raise the question how TRPML1 might influence transcriptional expression of E-cadherin.

As mentioned, past studies have shown that the loss of E-cadherin levels promote the activation of NF-κB and consequently the expression of E-cadherin suppressors, that is, snail, slug, and ZEB1/2 (48, 49, 50, 51, 52). In this regard we further investigated the effect that TRPML1 KO might have on tumor suppressor NF-κB. In most resting cells, NF-κB proteins exist in an inactive cytoplasmic state complexed to inhibitor-of-kappaB proteins. Exposure of external factors results in dissociation of NF-κB from inhibitor-of-kappaB, subsequently translocating to the nucleus and activating target genes. Alternatively, NF-κB activation can occur through phosphorylation of functional domains of NF-κB proteins themselves, namely the phosphorylation of p65 (80). Indeed, we observed an upregulation of phosphorylated-p65 protein levels and a significant downregulation of the inactive form p65 in RIL-175 KO cells (Fig. 7*G*), consequently promoting expression of E-cadherin suppressors snail and slug (Fig. 7, *E* and *F*).

Herein, our results indicate that a loss of E-cadherin levels in RIL-175 KO cells is accompanied by a reduction of colocalization with β-catenin (Fig. S3*B*). Also, β-catenin is found to be more represented in the cytosol and no longer at cell–cell contact site (Fig. 5*F*). Liberated β-catenin is known to accumulate in the cytoplasm and translocate to the nucleus, where it activates target genes (81, 82). Among them, β-catenin is known to activate p38, which in return phosphorylates p65 and therefore activates NF-κB. Consistent with previous results, we observed a significant downregulation of p38 and an upregulation of phosphorylated p38 (Fig. 7*H*). In contrary and in line with the previous observations, MDA-MB-231 cells display elevated β-catenin levels (Fig. 5*D*) and no expression of phosphorylated-p38 and therefore no p38-mediated NF-κB phosphorylation (Fig. 7*I*). Thus, we suggest that KO of TRPML1 impairs trafficking and consequently regulates E-cadherin expression on transcriptional level *via* β-catenin/p38/p65 axis in a cell line–dependant manner.

Overall, we propose that the opposing effects for our investigated cell lines might arise from tissue-specific functions of TRPML1. Indeed, TRPML1 is known to exhibit opposing functions in tumorigenesis as it may either be upregulated or downregulated in breast cancer or glioblastoma, respectively (34, 83). Additionally, TRPML1 has been implicated to have contrasting roles in autophagy regulation. While pharmacological channel activation hampers autophagy in HeLa (84), inhibition of TPRML1 with EDME in MDA-MB-231 equally attenuates autophagy (34). In line, we have observed opposing protein expression of EEs and recycling endosomes markers Rab5 and Rab11 (28) and a stronger colocalization of TRPML1 with Rab11 than with Rab5 in RIL-175 cells, suggesting a cell line–dependent effect on intracellular trafficking processes.

In conclusion, we demonstrate that loss of TRPML1 function leads to disturbed trafficking of important regulators of migration, that is, E-cadherin, β1-integrin, and cytoskeleton organization resulting in reduced migration and adhesion of cancer cells *in vitro* and reduced dissemination *in vivo.* Furthermore, the loss of E-cadherin levels at membrane site lead to a p38-mediated activation (RIL-175) or inhibition (MDA-MB-231) of NF-κB and therefore form a transcriptional feedback for E-cadherin expression. Our study reveals the pivotal role for TRPML1 to fundamental processes in cancer cell migration, providing it to be an attractive target for the treatment of invasive cancers.

## Experimental procedures

### Cell lines and culture

RIL-175 cells were provided by Prof. Simon Rothenfußer (CIPS-M, LMU Munich) (85). RIL-175 KO cells were generated by our group (32). MDA-MB-231 and HepG2 cells were obtained from DSMZ (#ACC 732, # ACC 180), Huh-7 from the Japanese Collection of Research Biorecourses and American Type Culture Collection (Japanese Collection of Research) and Hep3B cells from the Leibniz Institute. MDA-MB-231 KO, SkMel-5 WT, and SkMel-5 KO cells were provided by Prof. Christian Grimm (Walter-Straub-Institute of Pharmacology and Toxicology) (34). RIL-175, Huh-7, HepG2, SkMel-5, MDA-MB-231, and SkBr-3 were cultivated in Dulbecco’s modified Eagle’s medium (DMEM) (Anprotec, #AC-LM-0012) supplemented with 10% fetal calf serum (FCS) (Anprotec, #AC-SM-0027) at 37 °C, 5% CO_2_. For B16F10-luc RPMI 1640 medium (PAN-Biotech, P04-18500) and for Hep3B minimum essential medium Eagle (PAN-Biotech, P04-08500) medium each supplemented with 10% FCS was used. None of the cell lines used is listed in the database of commonly misidentified cell lines maintained by International Cell Line Authentication Committee. All cells are proven to be mycoplasma-free quarterly.

### Generation of a TRPML1 CRISPR/Cas9 KO cell line

The TRPML1 KO cell line in murine B16F10-luc cells was conducted with the CRISPR-Cas9 system as described earlier (86). To do so, we deleted exon 2 of the *MCOLN1* gene. Then, single-guide RNAs used by Siow *et al* (2023) were cloned into the pSpCas9(BB)-2A-GFP (PX458) plasmid (Addgene, #48138). The plasmids were transformed into competent DH5α-*Escherichia coli* and subsequently prepared using the QIAGEN Plasmid Maxiprep Kit according to the manufacturer’s instructions. We then confirmed correct insertions by sequencing from the U6 promotor. After successful confirmation, B16F10-luc WT cells were transfected with both plasmids according to the Lipofectamine 3000 (Invitrogen) manufacturer’s instructions followed by single-cell sorting (Cell Sorter BD FACSAria Fusion) into 96-well plates and subsequent clonal expansion. Successful exon 2 deletion was confirmed by standard PCR (Thermo Scientific Phusion Green Hot Start II High-Fidelity Polymerase, Thermo Fisher Scientific), agarose gel analysis, and Sanger sequencing.

### Generation of KD cells with siRNA

TRPML1 and Rab11A/B siRNA KDs in HepG2, Hep3B, Huh-7, and SkBr-3 WT cells were generated to further investigate the effects after the reduction of TRPML1 expression. For this purpose, cells were seeded in a 6-well plate 24 h prior to transfection. On the day of transfection, the culture medium was replaced with 2 ml of prewarmed OptiMEM-medium just before the addition of the siRNA–lipid complexes. The siRNA–lipid complexes were prepared according to the Thermo Fisher manufacturer’s protocol Lipofectamine RNAiMAX (Cat#: 13778-100) and siRNAs at a total final concentration of 20 nM (nontargeted siRNA, MCOLN-1 (h) siRNA, (h) Rab11A, (h) Rab11B). WT cell line treated with nontargeted siRNA served as control in all experiments and are referred to as WT in all figures for simplicity. After addition of siRNA–lipid complexes to the cells, they were left for incubation for 48 h and KD efficiency was determined *via* real-time quantitative PCR. Actin served as a housekeeping gene.

### *In vivo* experiments

All animal experiments were approved by the District Government of Upper Bavaria, in accordance with institutional guidelines and the German Animal Welfare. Mice were kept in a temperature-controlled facility with a usual 12 h/12 h light and dark schedule. Humidity was kept at 60% and the room temperature (RT) at 22 °C, both of which were continuously monitored by operating technology. Light intensity in the facility was kept at around 120 lux, in the racks at around 40 to 60 lux. Mice were maintained in a group of five in cages that can be ventilated individually, and which had an area of 700 cm2 (IVC, type 2 long, System Techniplast).

In total, 40 C57Bl/6-Tyr mice (Envigo), female, 5 weeks old, were used for intravenous injection of 200,000 B16F10-luc/RIL-175 WT or TRPML1 KO cells into the tail vein. Bioluminescence images were conducted using the IVIS Lumina system (PerkinElmer) on day 1, 4, 6, 8, 11, and 13 after intraperitoneal injection of 6 mg/ml luciferin per mouse. Previously, mice were put under anesthesia with 2.5% isoflurane in oxygen and mice were kept under narcosis with 1.5% isoflurane in oxygen. Hypothermia was prevented by a heating plate (37 °C). The tumor signal per defined region of interest was calculated as photons/second/cm (total flux/area) using the Living Image 4.7.4 software (PerkinElmer; https://www.perkinelmer.com/de/lab-products-and-services/resources/in-vivo-imaging-software-downloads.html#LivingImage).

### Migration assays

The wound-healing assay (87), Boyden-Chamber assay (70), and the circular micropatterning (88) were performed as recently described.

For spheroid migration, 20 μl drops of a cell suspension containing 50·10^3^ RIL-175 cells/ml and 20% methocel stock solution (1.2% (w/v) autoclaved methylcellulose (Sigma-Aldrich, #M0152) in ECGM (PeloBiotech, #PB-MH-100-2199)) were pipetted onto the lids of a 10 cm petri-dish. The lid was placed back onto the petri-dish and incubated for 24 h, allowing spheroid formation (hanging drop method).

Spheroids were embedded in TeloCol-6 collagen type I neutralized 1:10 with the supplied neutralization solution (Advanced Biomatrix, #5225, #5229) and diluted to 2.1 mg/ml in PBS. An 8-well μ-slide (ibidi) was coated with a thin base layer containing neutralized TeloCol-6:DMEM (2.125:1) on ice before incubation (30 min, 37 °C, 5% CO_2_). Spheroid-containing drops were washed down using PBS, centrifuged (1000 rpm, 5 min, 25 °C), and resuspended in 200 μl of FCS. EDME (50 μM) (34) was applied onto the base layer, then 125 μl of a mixture containing neutralized TeloCol-6:spheroids in FCS (2.125:1) were placed on top of the base layer. After an incubation time of 30 min (37 °C, 5% CO_2_), 100 μl of DMEM were added. Spheroids were incubated for 48 h and then imaged using a Leica Dmi1 inverted microscope equipped with a MC120HD camera (Leica). Spheroid diameters and area were determined by ImageJ (NIH; https://imagej.net/ij/download.html).

### Proteome analysis

Cells were lysed in 8 M Urea/50 mM NH4HCO3 in water using ultrasonication (Sonopuls GM3200, BR30, Bandelin). Protein concentration was determined using a Pierce 660 nm assay (Thermo Fisher Scientific). Ten micrograms of total protein was reduced with DTT (4 mM final concentration) and Tris(2-carboxyethyl)phosphine hydrochloride (2 mM final concentration) for 30 min at 56 °C and alkylated in the dark at ambient for 30 min using iodoacetamide (8 mM final concentration). Samples were digested with LysC (1:100 enzyme:protein) for 4 h at 37 °C, diluted with 50 mM NH_4_HCO_3_ in water to 1 M urea, trypsin was added (1:50 enzyme:protein) and samples were digested for 18 h at 37 °C.

For mass spectrometry (MS) analysis, an Ultimate 3000 nanochromatography system (Thermo Fisher Scientific) coupled to a Q Exactive HF X mass spectrometer (Thermo Fisher Scientific) was used. For each sample, 1 μg of peptides were injected and separated at 250 nl/min using an Easy-Spray column (PepMap RSLC C18 2 μm 100 Å 75 μm × 50 cm, Thermo Fisher Scientific) with the following eluents: 0.1% formic acid in water as eluent A and 0.1% formic acid in acetonitrile as eluent B. The separation method consisted of an initial ramp from 3% eluent B to 6% in 1 min, followed by an 80 min gradient to 20% and finished with a 9 min gradient to 40%. MS spectra were acquired with a top 15 data-dependent method. For protein identification, MaxQuant (v. 2.0.3.0; https://www.maxquant.org/) (89) and the murine subset of the UniProtKB/Swiss-Prot database was used. Data analysis was performed using Perseus (90), R (http://www.R-project.org/regarding), and the R packages (https://maxquant.net/perseus/) from tidyverse (https://github.com/tidyverse/tidyr; https://tidyr.tidyverse.org).

For GSEA, the label-free quantification data was log2 transformed, filtered for at least four valid values in at least one condition, and missing values were imputed from a normal distribution (width = 0.3; down shift = 1.8). The values were detransformed and loaded into the GSEA software ShinyGO 0.76 (http://bioinformatics.sdstate.edu/go76/) (91). As a pathway database “GO biological process” was selected, gene names were used without collapsing. The number of permutations was 10,000. All mouse gene ontologies were used as gene sets database.

### Real-time quantitative PCR

Real-time quantitative PCR was performed as described recently (92). The relative gene expression was normalized against housekeepers’ actin or tubulin for MDA-MB-231 and calculated as a fold-change compared to the WT cells using the ΔΔC_T_ method (93). Primers (Tables S1 and S2) were purchased from Metabion and validated for their specificity and efficiency prior to use.

### Western blot analysis

Western blot analysis was performed as previously described (92) using primary antibodies and secondary Horseradish peroxidase-coupled antibodies listed in Table S3. Chemiluminescence was detected on a Chemidoc Touch Imaging System (Bio-Rad). Data was processed with ImageLab (Bio-Rad) and normalized to total protein (stain-free detection) (94).

For the compound stimulation cells were grown in 6-well plates and treated as indicated (24 h) with chloroquine (Sigma-Aldrich, #C6628) or EDME. For Rab11-overexpression cells were transiently transfected with pEGFP-C1*-*Rab11-WT (Addgene, #12674) or pmaxGFP-plasmid (Lonza, #VCA-1001) using the Lipofectamine 3000 transfection reagent (Thermo Fisher Scientific, #100022057) according to manufacturer’s instructions and incubated (48 h). For cathepsin B release cells were grown overnight, medium was exchanged to FCS-free medium, and treated with ionomycin (5 μM) (Sigma, #10634) for 10 min (95). Supernatant was concentrated using Merck Amicon centrifugal units (Thermo Fisher Scientific, #10341782).

### Confocal microscopy

All confocal images were collected on a Leica SP8 inverted scanning microscope (Leica). Cells were grown in collagen-coated 8-well μ-slides (ibidi) overnight. Cells were fixed (MeOH, 10 min, RT) and permeabilized (acetone, 1 min, on ice). Unspecific binding sites were blocked with 5% bovine serum albumin in PBS (1 h, RT). After incubation with primary antibodies (overnight, 4 °C) and secondary antibodies (1 h, RT), cells were mounted with FluorSave mounting medium (Merck Millipore, #345789), covered with glass cover slips, and imaged. Antibodies are listed in Table S3. Nuclei were stained with Hoechst 33,342 (Sigma-Aldrich) and actin with Rhodamine Phalloidin (Thermo Fisher Scientific).

For TRPML1-HA or Rab11-OE experiments, cells were transiently transfected with a pcDNA3.1-TRPML1-HA plasmid (Addgene, #18825) or pEGFP-C1*-*Rab11-WT plasmid (Addgene, #12674) using the Lipofectamine 3000 transfection reagent (Thermo Fisher Scientific) according to the manufacturer’s instructions. After 48 h, cells were fixed (MeOH, 10 min, RT), permeabilized (acetone, 1 min, on ice), and stained with the antibodies listed in Table S3. Hoechst 33,342 (Sigma-Aldrich, colocalization) or ToPo-3 iodide (Invitrogen, #T3605, Rab11-overexpression) was used for the nucleus staining.

For the adhesion assay, the protocol was adapted from (70). A 24-well plate was coated with collagen (Matrix Bioscience, #50104, 0.4% in PBS), fibronectin (R&D Systems, #1030-FN-01M, 10 μg/ml in PBS), or laminin (R&D Systems, #3446-005-01, 10 μg/ml in PBS) (1 h, 37 °C). Unspecific binding sites were blocked with 3% (w/v) BSA in PBS (30 min, 37 °C). Cells were seeded and allowed to adhere for 1 h. Cells were fixed (4% paraformaldehyde [Thermo Fisher Scientific, #38908], 20 min, RT), stained with rhodamine-phalloidin (Sigma-Aldrich, #R415) and Hoechst 33,342 (Sigma-Aldrich) (30 min), mounted with FluorSave Reagent (Merck Millipore), and imaged. Adhered cells were counted using ImageJ.

For staining of migrating cells, a confluent cell layer was scratched. Cells were allowed to migrate (5 h) and fixed with MeOH (10 min, RT, [antibodies]) or 4% paraformaldehyde (20 min, RT, [actin]). Antibodies are listed in Table S3. Rhodamine-phalloidin (Sigma-Aldrich) was used to stain actin, Hoechst 33342 (Sigma-Aldrich) for nuclei.

For FRAP-experiments, cells were grown in collagen-coated 8-well μ-slides (ibidi) overnight and transiently transfected with pcDNA3.1*-*E-cadherin-GFP (Addgene, #28009) using FuGENE HD transfection reagent (Promega Cooperation, #E2311) according to the manufacturer’s instructions. After 24 h, the FRAP-experiment was performed under constant humidity provided by an objective heater (Okolab). Employing the FRAP-tool on the LAS X Core Software (Leica; https://www.leica-microsystems.com/products/microscope-software/p/leica-las-x-ls/downloads/), photobleaching of a defined region of interest was performed by nine scanning iterations with a laser intensity of 100%. One prebleach and 15 postbleach images (10 iterations, 30 s intervals/five iterations, 60 s intervals) at lower laser intensities were collected. Intensities were measured by the FRAP tool of the Leica LASX software. Recovery half-times were calculated after exponential curve fitting.

For FITC-Dextran uptake, cells were grown overnight in collagen-coated 8-well μ-slides (ibidi) overnight, incubated with 200 μg/ml FITC-dextran (20 kDa) (Sigma-Aldrich, #FD20S) (2 h) and Hoechst 33,342 (Sigma-Aldrich) (15 min). After fixation (MeOH, 10 min, RT), samples were mounted with FluorSave mounting medium (Merck Millipore), covered with glass cover slips and imaged.

### Flow cytometry

#### Uptake of FITC-dextran

Cells were grown in 12-well plates overnight and incubated with 200 μg/ml FITC-dextran (20 kDa) (Sigma-Aldrich) as indicated. After trypsinization, cells were collected by centrifugation, washed, and resuspended in PBS. Flow cytometry experiments were performed on a BD fluorescence-activated cell sorting Canto II (BD Biosciences). Fluorescence intensity of FITC-dextran was analyzed using the FITC channel. Data was evaluated using FlowJo 7.6 (https://www.flowjo.com/solutions/flowjo/downloads).

#### Detection of cytosolic calcium

To determine cytosolic calcium levels cells were seeded into a 24-well plate and left for incubation overnight. On the next day the calcium sensitive dye Cal-520 AM was added to the cells at a final concentration of 1 μM per well. Then, cells were left for incubation for 2 h at 37 °C and 5% CO_2_ and afterward for 30 min at RT and light exclusion. After incubation medium was discarded, cells were washed once with prewarmed Hanks and Hepes buffer (Table S4), trypsinized, collected and transferred to fluorescence-activated cell sorting tubes. After washing and centrifuging, cells were resuspended in Hanks and Hepes buffer and immediately measured by flow cytometry.

### β-Hexosaminidase release

The β-hexosaminidase assay was performed as described recently (95).

### Release of FITC-dextran

Cells were seeded into 96-well plates and stimulated with 200 μg/ml FITC-dextran (20 kDa) (Sigma-Aldrich). After 24 h, cells were washed and incubated with 50 mM CaCl_2_ in phenol red free DMEM (Pan Biotech) as indicated. After diluting 1:10 in PBS, fluorescence intensity was measured using the Infinite 200 Pro Tecan Plate reader (Tecan Trading AG) (485/535 Ex/Em).

### β_1_-Integrin internalization assay

β_1_-integrin internalization was conducted as described recently (70).

### Statistical analysis

All experiments were conducted at least three times independently unless stated otherwise. Data represents mean ± SD. For quantification of images, at least 60 individual cells have been analyzed per biological replicate using ImageJ. Unless stated otherwise, statistical significance was determined with an unpaired *t* test with Welch’s correction using GraphPad Prism 9.3.0 (https://www.graphpad.com/features). Results were considered significant for *p* < 0.0332.

## Data availability

All data generated or analyzed during this study is included in this published article and its supplementary information files. Any additional information might be obtained from the corresponding author upon reasonable request. The MS proteomics data have been deposited to the ProteomeXchange Consortium *via* the PRIDE (96) partner repository with the dataset identifier PXD046212.

## Supporting information

This article contains supporting information.

## Conflict of interest

The authors declare that they have no conflicts of interest with the contents of this article.
