# Supplementary material for: Endolysosomal TRPML1 channel regulates cancer cell migration by altering intracellular trafficking of E-cadherin and β1-integrin
Source: J Biol Chem. 2023 Dec 21;300(1):105581. doi: 10.1016/j.jbc.2023.105581 (PMC10825694; doi:10.1016/j.jbc.2023.105581)
Supplement: Supplemental information [file mmc1.docx]

**Endolysosomal TRPML1 channel altering cancer cell migration by facilitating the intracellular trafficking of E-cadherin and β_1_-integrin**

***– Supporting Information –***

Nadine Frey^1,#^, Lina Ouologuem^1,#^, Julia Blenninger^1^, Wei-Xiong Siow^1^, Julia Thorn-Seshold^2^, Jan Stöckl^3^, Carla Abrahamian^4^, Thomas Fröhlich^3^, Angelika M. Vollmar^1^, Christian Grimm^4^, Karin Bartel^1,*^

^1^ Department of Pharmacy, Pharmaceutical Biology, Ludwig-Maximilians-University Munich, 81377 Munich, Germany

^2^ Department of Pharmacy, Ludwig-Maximilians-University Munich, 81377 Munich, Germany

^3^ Gene Center, Laboratory for Functional Genome Analysis, Ludwig Maximilians-University Munich, 81377 Munich, Germany

^4^ Walther-Straub-Institute of Pharmacology and Toxicology, Ludwig-Maximilians-University Munich, 80336 Munich, Germany

^#^ These authors contributed equally.

* Correspondence: [karin.bartel@cup.uni-muenchen.de](mailto:karin.bartel@cup.uni-muenchen.de), Twitter: @lysocancerlab

**Video S1: Loss of TRPML1 function leads to a reduction of collective cell migration.**

Wound healing assay of RIL-175 for 12 h **(A)** and MDA-MB-231 for 4.6 h **(B)** was performed and monitored via live-cell imaging technique. Left panel WT cells, right panel KO cells. Upon loss of TRPML1 function cells migration velocity is reduced.

**Figure S1: Loss of TRPML1 function hampers migration.**

Wound healing assay FCS controls. Scale bars 1mm. **(B)** siRNA knockdown efficiency evaluated by RT-qPCR **(C)** Spheroids of RIL-175 cells with or without EDME-stimulation (50 µM, 48 h). Analysis of longitudinal and transverse diameter and spheroid area by ImageJ suggests decreased migration upon TRPML1-inhibition. Scale bars 100µm. **(E)** PCR and agarose gel of potential KO clones using exon 2 spanning primers (Table S3). Clone 2A8 identified as potential clones. Thermo Scientific™ GeneRuler 1 kb Plus DNA-Ladder was used. **(F)** Following Sanger Sequencing and Two-Sequence alignment displayed missing exon2 in clone 2A8. Statistical significance was assessed by unpaired student’s t-test. * p < 0.0332, *** p < 0.0002, **** p < 0.001, ns= not significant.


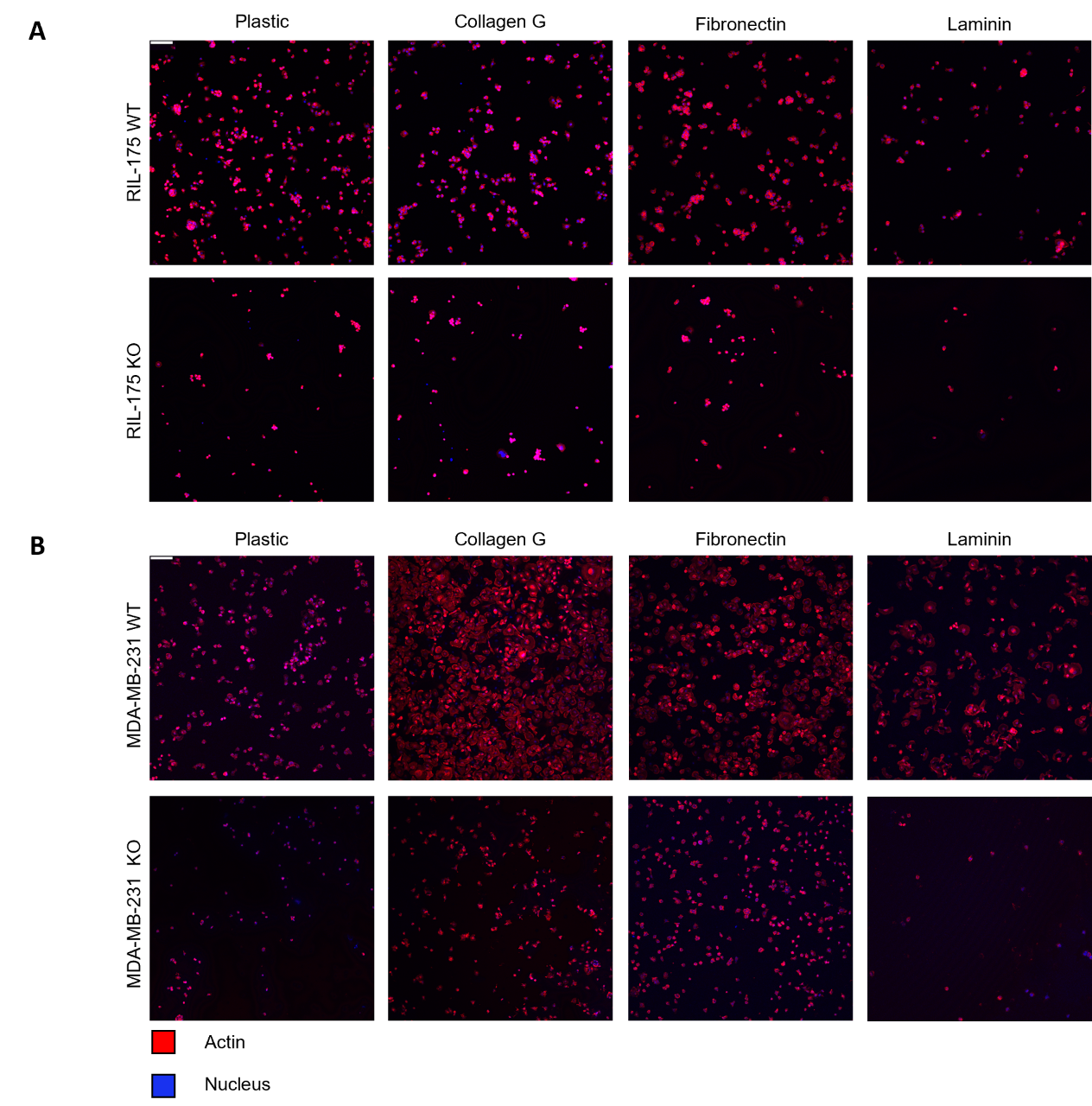


**Figure S2: Representative confocal images for the adhesion assay.**

Representative confocal images of adhered RIL-175 **(A)** and MDA-MB-231 **(B)** WT and TRPML1 KO cells stained for the actin cytoskeleton (red) and the nucleus (Hoechst). Scale bar 100 μm.


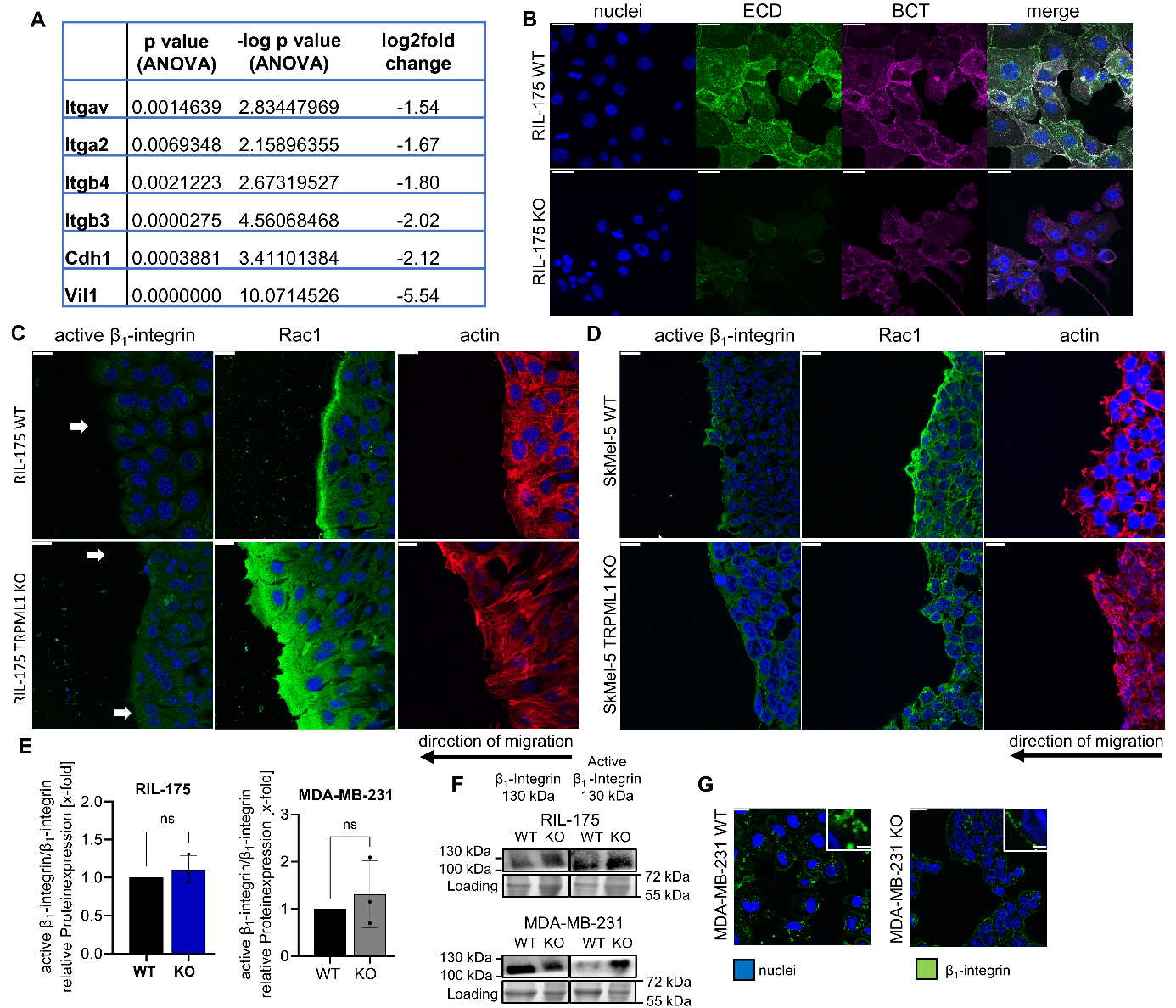


**Figure S3: Colocalization of E-cadherin and β-catenin in RIL-175 WT and KO cells.**

**(A)** Proteins significantly altered in TRPML1 KO cells and are being investigated in the following experiments. Data from Proteome analysis **(B)** Representative confocal images of RIL-175 WT and KO staining of E-cadherin, β-catenin, and nuclei. Scale bar 20µm. **(C, D)** After 5 h of migration time, cells were stained for active β_1_-integrin (left panel, green), Rac1 (middle panel, green), actin (right panel, red), and the nucleus (Hoechst). **(E, F)** Relative protein levels of active β_1_-intergrin in RIL-175 and MDA-MB-231 WT and KO cells. Blots for β_1_-integrin were generated on the same membrane as for Rac1 (RIL-175) or Rac1/Src (MDA-MB-231) in Figure 4, therefore LC have been reused and are identical for these proteins. **(G)** β_1_-intergrin in vesicles in MDA-MB-231 WT and KO cells. Images/Blots are representative. Scale bar 20µm. Scale bar zoomed pictures 2µm. Statistical analysis was was assessed by unpaired student’s t-test. ns= not significant.


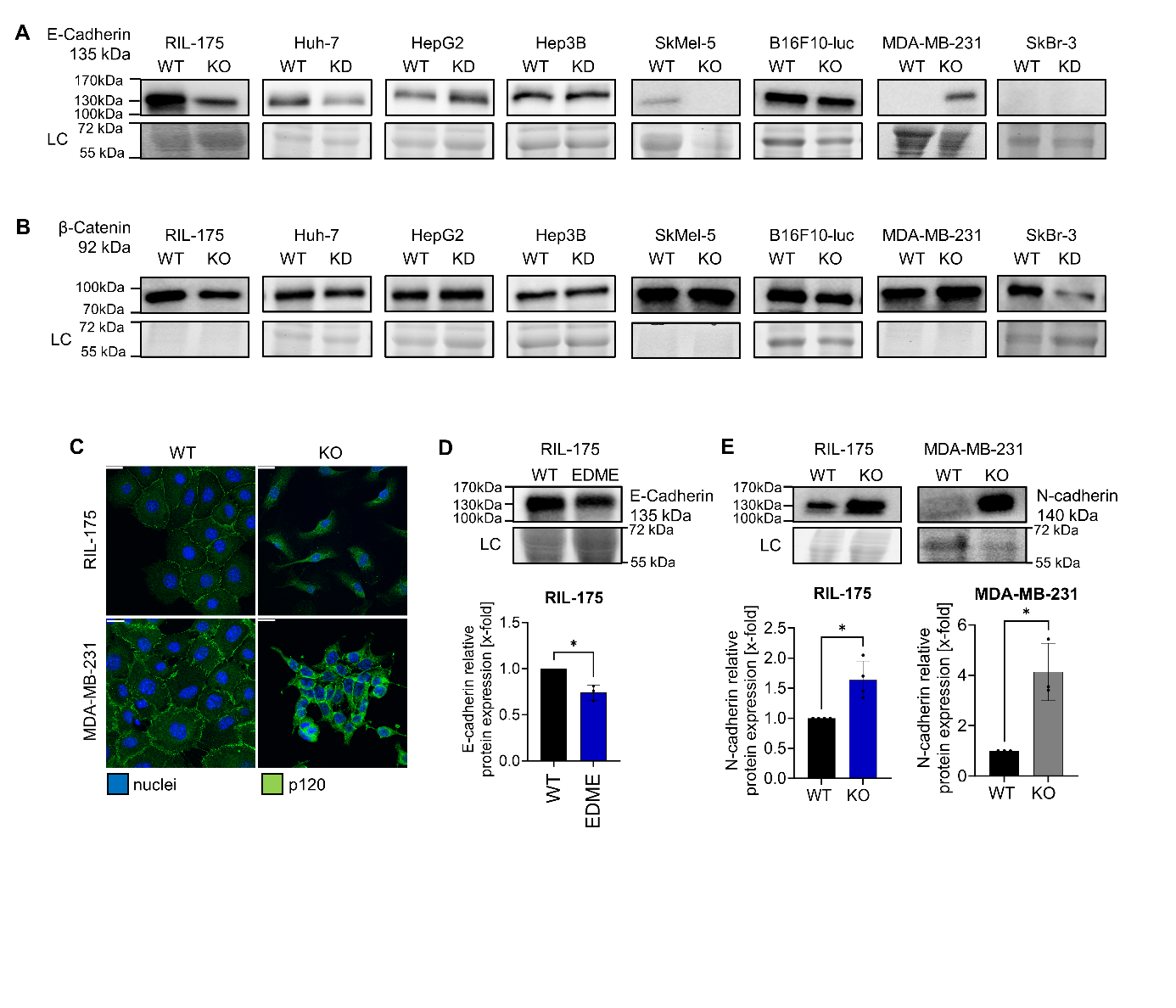


**Figure S4: Expression of adherence junction proteins.**

Relative protein levels of E-cadherin **(A)** and β-catenin **(B)**. Loading images for Huh-7, HepG2, Hep3B have been reused as proteins were blotted on the same membrane, due to different protein size, therefore LC are identical for those proteins. Loading control = LC. **(C)** Representative confocal images of RIL-175 and MDA-MB-231 WT and KO cells stained for p120 and nuclei. **(D)** Relative protein levels of E-cadherin in RIL-175 WT cells after treatment with 30µM EDME. **(E)** Relative protein levels of N-cadherin in RIL-175 and MDA-MB-231 WT and KO cells. Images/Blots are representative. Loading Control = LC. Scale bar 20 µm. Statistical significance was assessed by unpaired student’s t-test. * p < 0.0332.


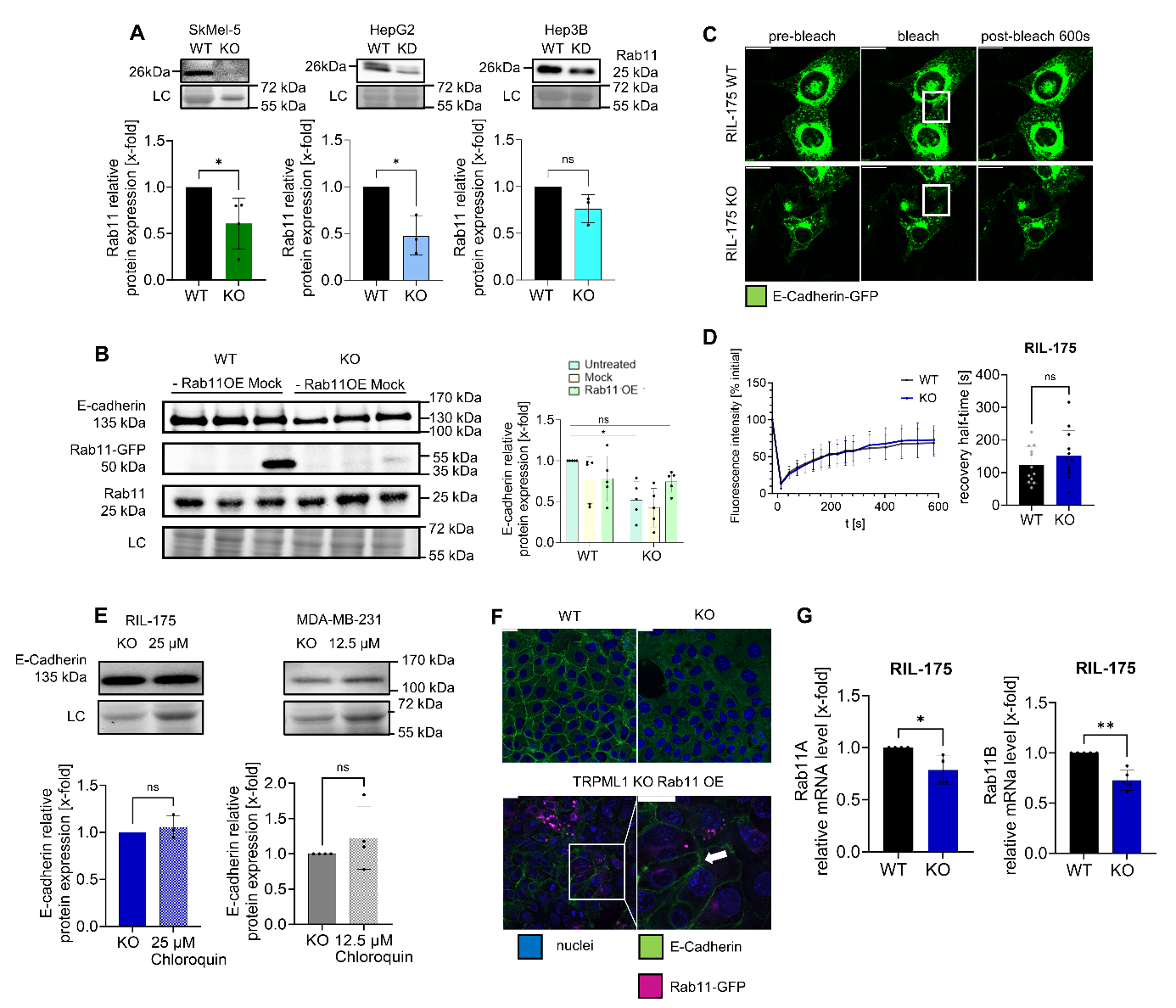


**Figure S5: Effects of TRPML1 KO on Rab-proteins.**

**(A)** Relative protein levels of Rab11 in SkMel-5, HepG2 and Hep3B WT and TRPML1 KO cells. **(B)** Relative protein levels of E-cadherin, Rab11-GFP, and endogenous Rab11 in whole cell lysates of Rab11-overexpressing (OE) RIL-175 WT and KO cells. An EGFP-plasmid served as negative control. **(C, D)** Representative confocal images of three FRAP-steps taken for RIL-175 WT and KO cells expressing E-cadherin-GFP (green). The boxes indicate the bleaching areas. E-cadherin recovery is blotted over time (left panel) and quantified as the recovery half-time (right panel). Scale bar 5µm. **(E)** Relative protein levels of E-cadherin in RIL-175 KO and MDA-MB-231 KO cells after 24 h stimulation with chloroquine 25 μM (RIL-175) or 12.5 μM (MDA-MB-231). **(F)** RIL-175 WT, KO, and Rab11-OE KO cells stained for E-cadherin, Rab11, and the nuclei. Scale bar 20µm. **(G)** mRNA levels of Rab11A and Rab11B in RIL-175 WT and TRPML1 KO cells acquired by RT-qPCR experiments. Statistical significance was assessed by unpaired student’s t-test with Welch’s correction. Loading control in Western Blots = LC. * p < 0.0332, ** p < 0.0021, ns= not significant (B) or by two-way ANOVA followed by Tukey’s multiple comparison test, * p < 0.0332 (B).


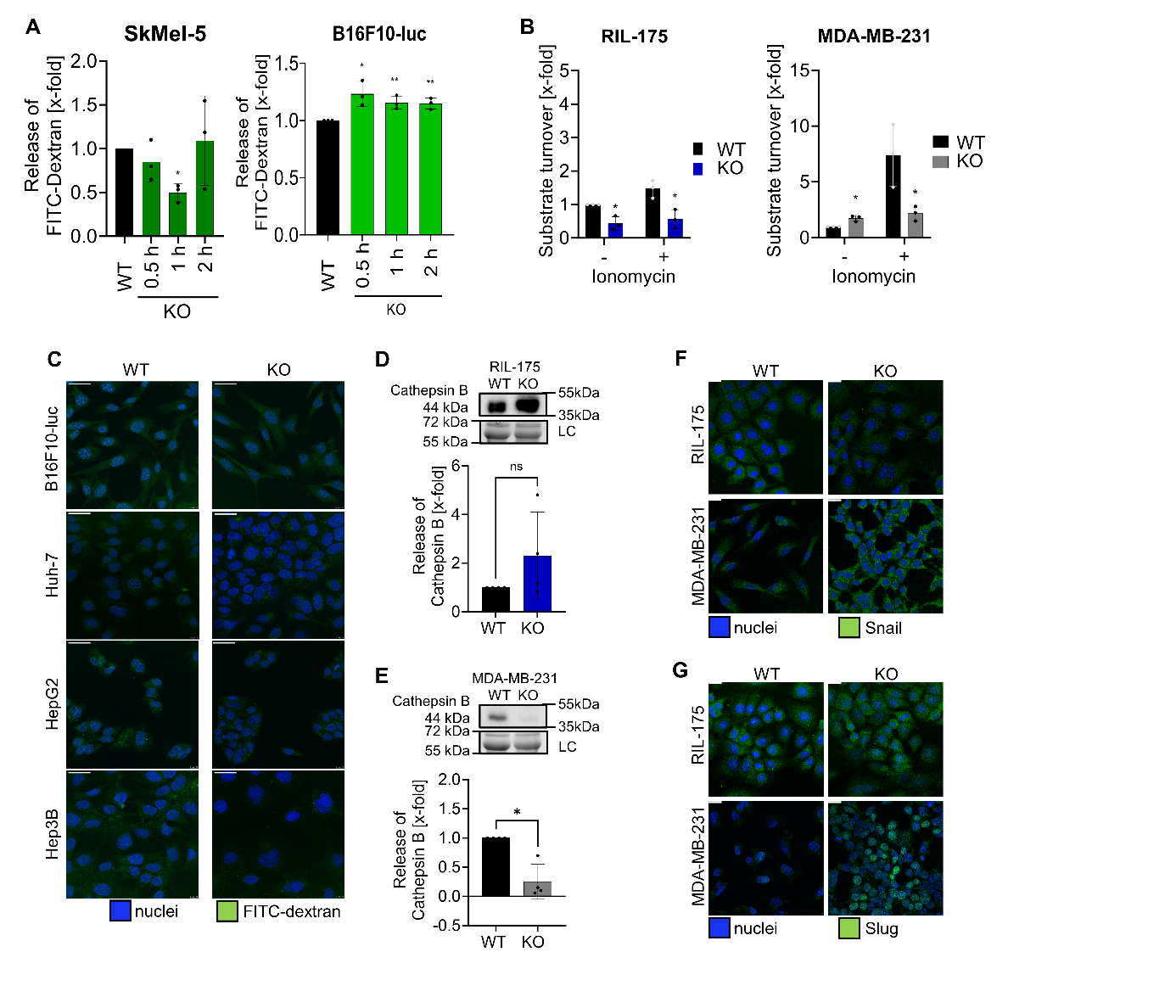


**Figure S6: Impaired trafficking upon TRPML1 loss of function.**

**(A)** Lysosomal exocytosis assay of released FITC-dextran (200 µg/mL, incubated for 24 h) upon calcium treatment (50 mM). The results were normalized to the WT level**. (B)** Lysosomal exocytosis assay showing decreased relative β-hexosaminidase release upon Ionomycin treatment (5 μM, 10 min). **(C)** Representative confocal images of B16F10-luc, Huh-7, HepG2 and Hep3B after 2h incubation with FITC-dextran (200µg/mL). **(D, E)** Relative protein levels of released Cathepsin B after Ionomycin treatment (5 μM, 10 min). Loading Control = LC. Representative confocal images of RIL-175 and MDA-MB-231 WT and KO cells stained for Snail **(F)** and Slug **(G).** Images/Blots are representative. Scale bar 20µm. Statistical significance was assessed by unpaired student’s t-test. * p < 0.0332, ** p < 0.0021, ns= not significant

| Target | Forward sequence (5’-3’) | Reverse sequence (5’-3’) |
| --- | --- | --- |
| E-cadherin | CAGAAAGTTTTCCACCAAAG | AAATGTGAGCAATTCTGCTT |
| β-catenin | AGGATACAGCGGCTTCTGCG | ACGCATGATAGCGTGTCTG |
| Rab11A | ACACGGAAGGCATTGCTGTA | GCACCCAAAGCACTCAAAGG |
| Rab11B | CAACCTGGAGAGCAAGAGCA | CAACCTGGAGAGCAAGAGCA |
| Tubulin α-1A | CACCATCAAAACCAAGCGCA | GCACGCTTGGCATACATCAG |
| Actin | CCAACCGCGAGAAGATGA | CCAGAGGCGTACAGGGATAG |

**Table S1: Primers for RT-qPCR analysis (human).**

| Target | Forward sequence (5’-3’) | Reverse sequence (5’-3’) |
| --- | --- | --- |
| Actin | CCACCATGTACCCAGGCATT | AGGGTGTAAAACGCAGCTCA |
| E-cadherin | GGTTTTCTACAGCATCACCG | GCTTCCCCATTTGATGACAC |
| β-catenin | GGCGGCCGCGAGGTA | TTAGTGGGATGAGCAGCGTC |
| Rab11A | GCCATTGTAGGGCCCTGAAA | CCCCACACATCATGAGCACT |
| Rab11B | TCCACCAAATGATGCACCTGA | GAGCTGACATCACCCACTGT |

**Table S2: Primers for RT-qPCR analysis (murine).**

| Target | Forward sequence (5’-3’) | Reverse sequence (5’-3’) |
| --- | --- | --- |
| Exon2 spanning primer pair | ACAGAATCCTAGACTGGCCT | AAGGTGGGTACAGGAGTGGT |

**Table S3: Exon spanning primers used for CRISPR/Cas9 KO validation.**

| Target | Tested Reactivity | Supplier |
| --- | --- | --- |
| Active β_1_-integrin | Human | Abcam, Cambridge, UK, #30394 |
| β-catenin | Human/Mouse | Santa Cruz, Dallas, USA, #7199 |
| E-cadherin | Human/Mouse | Cell Signaling Technologies (CST), Danvers, Massachusetts, #3195 |
| FAK | Human/Mouse | Santa Cruz, #1688 |
| β_1_-integrin | Human/Mouse | CST, #4706 |
| N-cadherin | Human/Mouse | CST, #4061 |
| p38 | Human/Mouse | CST, #9212 |
| phospho-p38 | Human/Mouse | CST, #9211 |
| p65 | Human/Mouse | Santa Cruz, sc-372-G |
| phosphor-p65 | Human/Mouse | CST, #3031 |
| p120-Catenin | Human/Mouse | Santa Cruz, #13957 |
| pFAK | Human/Mouse | Invitrogen, Karlsruhe, Germany, #44590G |
| pRac1 | Human/Mouse | Biomol, Hamburg, Germany, #26903 |
| pRhoA | Human | Calbiochem, Darmstadt, Germany #ST1035 |
| pSrc | Human/Mouse | CST, #6943 |
| Rab11 | Human/Mouse | Santa Cruz, #9020 or CST, #3539 |
| Rab5 | Human/Mouse | CST, #2143 |
| Rac1 | Human/Mouse | Sigma Aldrich, St. Louis, USA, #05-389 |
| RhoA | Human/Mouse | Santa Cruz, #418 |
| Slug | Human/Mouse | CST, #9585 |
| Snail | Human/Mouse | CST, #3879 |
| Src | Human/Mouse | Abcam, #47405 |
| ZO-1 | Human | CST, #8193 |
| Cathepsin B | Human/Mouse | CST, #31718 |
| HRP goat-anti rabbit | Goat anti Rabbit | Dianova,Hamburg,Germany,#111-035-144 |
| HRP goat-anti mouse IgG_1_ | Goat anti Mouse | Abcam, #97240 |
| Alexa Fluor 488 | Goat anti Rabbit | Invitrogen, #11008 |
| Alexa Fluor 488 | Goat anti Mouse | Invitrogen, #11001 |
| Alexa Fluor 546 highly cross-absorbed | Goat anti Rabbit | Invitrogen, #11035 |
| Alexa Fluor 680 | Goat anti Mouse | Invitrogen, # 21057 |
| HA-tag mAB | HA peptide | Invitrogen, #26183 |
| Alexa Fluor 546 highly cross-absorbed | Goat anti Rabbit | Invitrogen, #11035 |
| Alexa Fluor 405 | Goat anti Rabbit | Invitrogen, #31556 |
| Alexa Fluor 633 highly cross-absorbed | Goat anti Mouse | Invitrogen, #21050 |
|  |  |  |

**Table S4: Primary/Secondary antibodies for Western Blot and Immunofluorescence.**

| Chemical | Final concentration | Chemical | Final concentration |
| --- | --- | --- | --- |
| CaCl_2_ | 1.26 mM | MgCl_2_ x 6 H_2_O | 0.49 mM |
| D-glucose | 5.56 mM | MgSO_4_ x 7 H_2_O | 0.41 mM |
| Hepes | 20 mM | Na_2_HPO_4_ | 0.34 mM |
| KCl | 5.33 mM | NaCl | 137.9 mM |
| KH_2_PO_4_ | 0.44 mM | NaHCO_3_ | 4.17 MM |
|  |  |  | Ad H_2_O |

**Table S5: Hanks and Hepes Buffer (pH 7.2)**
